# GOLM1 depletion modifies cellular sphingolipid metabolism and adversely affects cell growth

**DOI:** 10.1016/j.jlr.2022.100259

**Published:** 2022-08-07

**Authors:** Meghana Nagaraj, Marcus Höring, Maria A. Ahonen, Van Dien Nguyen, You Zhou, Helena Vihinen, Eija Jokitalo, Gerhard Liebisch, P.A. Nidhina Haridas, Vesa M. Olkkonen

**Affiliations:** 1 Minerva Foundation Institute for Medical Research, Biomedicum, Helsinki, Finland; 2 Doctoral Programme in Integrative Life Science, University of Helsinki, Helsinki, Finland; 3 Institute of Clinical Chemistry and Laboratory Medicine, University Hospital Regensburg, Regensburg, Germany; 4 Doctoral Programme in Clinical Research, University of Helsinki, Helsinki, Finland; 5 Systems Immunity University Research Institute, and Division of Infection and Immunity, Cardiff University, Cardiff, United Kingdom; 6 Electron Microscopy Unit, Institute of Biotechnology, University of Helsinki, Helsinki, Finland; 7 Department of Anatomy, Faculty of Medicine, University of Helsinki, Helsinki, Finland

**Keywords:** ceramide, cholesteryl ester, glycosphingolipid, Golgi, GOLPH2, GP73, phosphatidylethanolamine, mitochondrial function, hexosylceramide, dihexosylceramide, ACBD3, acyl-CoA binding domain containing 3, CE, cholesteryl ester, Cer, ceramide, CerP, ceramide phosphate, FIA, flow injection analysis, GARP, Golgi-associated retrograde protein, GlcCer, glucosyl ceramide, GOLM1, Golgi membrane protein 1, GP73, Golgi phosphoprotein 73, GSL, glycosphingolipid, HBV, hepatitis B virus, HCC, hepatocellular carcinoma, HCV, hepatitis C virus, HexCer, hexosylceramide, IS, internal standard, LacCer, lactosylceramide, LPE, lysophosphatidylethanolamine, MEM, minimal essential medium, NAFLD, nonalcoholic fatty liver disease, NASH, nonalcoholic steatohepatitis, OCR, oxygen consumption rate, PE, phosphatidylethanolamine, qPCR, quantitative PCR, QQQ, triple quadrupole, SARS-CoV-2, severe acute respiratory syndrome coronavirus 2, VPS, vacuolar protein sorting

## Abstract

Golgi membrane protein 1 (GOLM1) is a Golgi-resident type 2 transmembrane protein known to be overexpressed in several cancers, including hepatocellular carcinoma (HCC), as well as in viral infections. However, the role of GOLM1 in lipid metabolism remains enigmatic. In this study, we employed siRNA-mediated GOLM1 depletion in Huh-7 HCC cells to study the role of GOLM1 in lipid metabolism. Mass spectrometric lipidomic analysis in GOLM1 knockdown cells showed an aberrant accumulation of sphingolipids, such as ceramides, hexosylceramides, dihexosylceramides, sphinganine, sphingosine, and ceramide phosphate, along with cholesteryl esters. Furthermore, we observed a reduction in phosphatidylethanolamines and lysophosphatidylethanolamines. In addition, Seahorse extracellular flux analysis indicated a reduction in mitochondrial oxygen consumption rate upon GOLM1 depletion. Finally, alterations in Golgi structure and distribution were observed both by electron microscopy imaging and immunofluorescence microscopy analysis. Importantly, we found that GOLM1 depletion also affected cell proliferation and cell cycle progression in Huh-7 HCC cells. The Golgi structural defects induced by GOLM1 reduction might potentially affect the trafficking of proteins and lipids leading to distorted intracellular lipid homeostasis, which may result in organelle dysfunction and altered cell growth. In conclusion, we demonstrate that GOLM1 depletion affects sphingolipid metabolism, mitochondrial function, Golgi structure, and proliferation of HCC cells.

Golgi membrane protein 1 (GOLM1), a Golgi type 2 transmembrane protein also known as Golgi phosphoprotein 73 (GP73) or Golgi phosphoprotein 2 (GOLPH2), is expressed primarily in epithelial cells, and it is overexpressed upon viral infections including severe acute respiratory syndrome coronavirus 2 (SARS-CoV-2), Wilson disease, and cancers (1, 2, 3, 4). GOLM1 expression is elevated in liver diseases, such as acute and autoimmune hepatitis, hepatitis B virus (HBV) and hepatitis C virus (HCV) infections, alcohol-related liver diseases, nonobese nonalcoholic fatty liver disease (NAFLD), and hepatocellular carcinoma (HCC) (5, 6, 7). *Golm1* is not expressed in healthy rat hepatocytes; however, its expression increases during malignant transformation (6) and contributes to HCC cell proliferation and metastasis (8). In addition, *Golm1* expression is also known to be induced in the liver of mice fed with high-fat and high-cholesterol cholate diet (7). Recent study also showed a positive correlation between hepatic *GOLM1* mRNA expression and nonalcoholic steatohepatitis (NASH) (9). GOLM1 enhances metastasis by acting as a cargo adaptor protein for epidermal growth factor receptor recycling and signaling (10). GOLM1 also regulates tumor microenvironment by suppressing CD8^+^ T cells and activating endoplasmic reticulum stress in tumor-associated macrophages (11, 12). Moreover, mammalian target of rapamycin complex 1 activation, starvation, and nutritional stimuli modulate GOLM1 expression (2, 13). Furthermore, GOLM1 is suggested to act as a gluconeogenic hormone in SARS-CoV-2 infection (2). GOLM1 was recently reported to possess Rab GTPase-activating protein activity and affects apolipoprotein B secretion (7). However, very little data are available on the role of GOLM1 in the lipid metabolism of hepatic cancer cells.

Human HCC is a complex and aggressive form of liver cancer with a high mortality rate, its incidence being four times higher in men than women (14, 15, 16). Several factors contribute to the etiology of HCC, including genetics, HBV and HCV infections, alcoholic fatty liver cirrhosis, NAFLDs, toxins, and carcinogenic exposure (17, 18, 19, 20, 21). Significant progress has been made in understanding the pathophysiology, diagnosis, and treatment of HCC and other cancers during the past decades. The roles of Golgi apparatus and its resident proteins in the progression of cancers are becoming increasingly evident (22, 23, 24). GOLM1/GP73 is one of the Golgi proteins involved in the pathogenesis of HCC (8, 25).

In this study, the effect of GOLM1 depletion on the lipid profile and metabolism was characterized in HCC cells. In addition, the changes in Golgi morphology, apoptosis, mitochondrial function, and proliferation in GOLM1-depleted cells were analyzed.

## Materials and methods

### Cell culture and transfections

Huh-7 and HepG2 HCC cell lines were cultured in Eagle's minimal essential medium (MEM), GlutaMAX™ Supplement (Gibco; Thermo Fisher Scientific, Inc, Waltham, MA; catalog no.: 41090-036), and MEM AQ™ (minimal essential Eagle's medium; Sigma-Aldrich, Merck, St Louis, MO; catalog no.: M0446) containing 10% FBS (Sigma-Aldrich; catalog no.: F9665), 100 U/ml penicillin, and 100 μg/ml streptomycin. Huh-7 cells were reverse transfected with 100 or 150 nM negative control siRNA (Invitrogen, Life Technologies Corp, Thermo Fisher Scientific, Inc, Pleasanton, CA; catalog no.: 4390847) or GOLM1 siRNA (Flexi tube siRNA; Qiagen, Hilden, Germany; catalog no.: SI03130904) using Lipofectamine™ RNAiMAX Transfection Reagent (Invitrogen, Life Technologies Corp, Thermo Fisher Scientific, Inc, Carlsbad, CA; catalog no.: 13778-150) according to the manufacturer's protocol. Depending on the experiments, the cells were transfected for 24–72 h and used for downstream analyses.

### Data mining

Two microarray datasets (GSE62232 and GSE164760) of HCC cohorts with different etiologies were downloaded from the Gene Expression Omnibus database (https://www.ncbi.nlm.nih.gov/geo/). The GSE62232 dataset included 22 tumors from alcohol-related etiology, 21 tumors with HBV or HCV as the etiology, and 10 HCC adjacent samples for examining viruses and alcohol-induced HCC (26). To assess HCC caused by NAFLD, we employed GSE164760 dataset with 170 samples (53 NASH-HCC tumors, 29 NASH-HCC adjacent samples, and 6 healthy controls) (27). Briefly, raw data were downloaded as CEL files and underwent robust multichip average (RNA) normalization using affy package (28). Subsequently, the dataset’s probe sets were annotated using their corresponding chips. The expression profile of GOLM1 was then extracted from the retrieved datasets for downstream comparative analyses.

### Immunofluorescence microscopy

Cells were cultured in 12- or 4-well plates on coverslips. Cells were washed two times with PBS and fixed with 4% paraformaldehyde (in PBS) for 20 min, followed by three washes with ice-cold PBS. Cells were then permeabilized using 0.1% Triton X-100 for 5 min. The fixed and permeabilized cells were blocked for 60 min using 10% FBS (in PBS) at room temperature. After blocking, the cells were incubated at 4°C overnight with the primary antibody, anti-GOLM1 (Novus Biologicals; catalog no.: NBP1-50627), and anti-Golgin subfamily A member 2 (anti-GM130; BD Transduction Laboratories™; catalog no.: 610822). The cells were washed three times with PBS for 5 min and probed with the respective secondary antibody (Alexa Fluor™ 647, Thermo Fisher Scientific, catalog no.: A-21244; Alexa Fluor™ 488, Thermo Fisher Scientific, catalog no.: A-11001) for 60 min at 37°C, followed by three washes with PBS. The cells were mounted with 50 mg/ml 1,4-diazabicyclo[2.2.2]octane (Sigma-Aldrich, Merck; catalog no.: D2522), Mowiol (Sigma-Aldrich, Merck; catalog no.: 475904-M) containing 5 μg/ml 4′,6-diamidino-2-phenylindole, dihydrochloride (Invitrogen, Thermo Fisher Scientific, Inc; catalog no.: D1306). Fluorescence was observed with a 63× oil objective in ZEISS LSM 880 with Airyscan at HiLIFE Biomedicum Imaging Unit, University of Helsinki. ImageJ (FIJI) software was used to quantify the cell number, Golgi stack distribution, and the length of the Golgi stack from the nucleus.

### Western blotting

GOLM1 protein expression was analyzed by Western blotting. After 72 h of silencing, control and GOLM1-silenced cells were lysed with RIPA buffer (15 mM Tris-HCl buffer, pH 7.4 containing 1% NP-40, 1.25% sodium deoxycholate, 150 mM NaCl, 1 mM EDTA, 1% SDS, Complete™, Mini, EDTA-free Protease Inhibitor Cocktail, [Roche Diagnostics GmbH, Mannheim, Germany; catalog no.: 04693159001]), and equal amount of protein was resolved on 10% or 12% SDS-polyacrylamide gels (Fast-Cast TGX Stain-free; Bio-Rad; catalog no.: 1610183), followed by transferring onto PVDF membrane using Bio-Rad Transblot system. The membrane was blocked to eliminate nonspecific antibody binding using 5% milk in TBS with 0.1% Tween for 60 min and probed with anti-GOLM1 (Novus Biologicals; catalog no.: NBP1-50627), anti-ORMDL3 (Novus Biologicals; catalog no.: NBP1-98511), and corresponding HRP-conjugated secondary antibodies. Signals were developed with Pierce™ ECL Western Blotting Substrate (Thermo Scientific™, Thermo Fisher Scientific, Inc; catalog no.: 32106) or Clarity™ Western ECL Substrate (Bio-Rad; catalog no.: 1705060) and captured using ChemiDoc™ Touch Gel Imaging System (Bio-Rad; catalog no.: 1708370). Protein expression was quantified using Image Lab™ Software (Bio-Rad) and is normalized to the total protein intensity of the blot lane.

### Quantitative real-time PCR

Total RNA from Huh-7 cells was extracted using PureLink ™ RNA Mini Kit (Invitrogen, Thermo Fisher Scientific, Inc; catalog no.: 12183018A) according to the manufacturer’s instruction, and RNA was reverse-transcribed into complementary DNA using SuperScript® VILO™ synthesis Kit (Invitrogen, Thermo Fisher Scientific, Inc; catalog no.: 11754-050). The mRNA expression of genes was analyzed by quantitative PCR (qPCR) using gene-specific primers and LightCycler® 480 SYBR Green (Roche Molecular Systems, Mannheim, Germany; catalog no.: 04887352001) master mix. The reactions were carried out using the LightCycler 480 II Real-Time PCR system (Roche Applied Science, Penzberg, Germany). The expression was calculated using the CT values and was normalized to the housekeeping gene expression (actin and succinate dehydrogenase complex, subunit A).

Mitochondrial DNA amount was measured using qPCR, and total DNA was isolated from GOLM1-depleted and control cells using genomic DNA isolation kit according to the manufacturer’s protocol (QIAamp DNA Mini Kit; Qiagen; catalog no.: 51304). qPCR was performed using mitochondrial and nuclear gene–specific primers. 2^(−ΔCT)^ was calculated for both nuclear and mitochondrial gene measurements; further, mitochondrial measurements were normalized to genomic measurements. *FABP1* and *RPLPO* were used as nuclear gene references. Primer sequences used are provided in the supplemental Table S1.

### Lipidomics

GOLM1 knockdown and control Huh-7 samples were subjected to quantitative lipid MS. Cell homogenates were extracted according to the method of Bligh and Dyer (29) in the presence of not naturally occurring lipid species as internal standards (ISs). The analysis of lipids was performed by direct flow injection analysis (FIA) using a triple quadrupole (QQQ) mass spectrometer (FIA-MS/MS; QQQ triple quadrupole) and a hybrid quadrupole-Orbitrap mass spectrometer (FIA-FTMS; high mass resolution).

FIA-MS/MS (QQQ) was performed in positive ion mode using the analytical setup and strategy described previously (30). A fragment ion of *m/z* 184 was used for phosphatidylcholine, SM, and lysophosphatidylcholine. The following neutral losses were applied: phosphatidylethanolamine (PE) and lysophosphatidylethanolamine (LPE) 141, phosphatidylserine 185, phosphatidylglycerol 189, and phosphatidylinositol 277 (31). PE-based plasmalogens were analyzed according to the principles described by Zemski Berry (32). Sphingosine-based ceramides (Cers) and hexosylceramides (HexCers) were analyzed using a fragment ion of *m/z* 264. Quantification was achieved by calibration lines generated by addition of naturally occurring lipid species to the respective sample matrix.

The FIA-FTMS setup is described in detail in the study by Höring *et al.* (33). Triglycerides, diglycerides, and cholesteryl esters (CEs) were recorded in positive ion mode FTMS at a target resolution of 140,000 (at *m/z* 200). Multiplexed acquisition (MSX) was used for the [M + NH_4_]^+^ of free cholesterol (*m/z* 404.39) and D7-cholesterol (*m/z* 411.43) (34). Data processing details were described in the study by Höring *et al.* (33) using the ALEX software, which includes peak assignment and intensity picking (35). FIA-FTMS quantification was performed by multiplication of the spiked IS amount with analyte-to-IS ratio.

Cell homogenates were extracted using butanol in the presence of non-naturally occurring ISs and subjected to hydrophilic interaction liquid chromatography coupled to MS/MS to quantify dihexosylceramides (Hex2Cer), sphingoid bases, and ceramide phosphate (CerP) (36).

The extracted data were exported to Microsoft Excel 2016 and further processed by self-programmed macros including type II and type I correction as described. Lipid species were annotated according to the latest proposal for shorthand notation of lipid structures that are derived from MS (37). For QQQ glycerophospholipid species, annotation was based on the assumption of even numbered carbon chains only. SM species annotation is based on the assumption that a sphingoid base with two hydroxyl groups is present.

### Total cholesterol enzymatic assay

GOLM1-silenced and control cells were scraped in 2% sodium chloride 72 h after the transfection, and lipids were Bligh and Dyer (29) extracted. After the isolation, the samples were analyzed with CHOD-PAP cholesterol reagent (Roche Diagnostics GmbH; catalog no.: 11491458216). The results were normalized to the total protein.

### TLC

GOLM1-silenced and control cells were grown in 6-well plates. At 72 h after transfection, the cells were labeled for 4 h in FBS-containing MEM with 20 μCi/well [^3^H] acetic acid (PerkinElmer, Waltham, MA; catalog no.: NET003025MC). The cells were washed twice with PBS and then scraped in 2% sodium chloride. TLC was performed after isolating the total lipids according to the extraction protocol by Bligh and Dyer (29). The samples were run on Merck silica plates (Supelco; catalog no.: 105721) with hexane:diethylether:acetic acid:water in the ratio 65:15:1:0.25 for [^3^H] acetic acid–labeled samples. To identify the lipids, cholesterol, CE, and triglyceride standards were run along with the samples. The corresponding bands were scratched and added to OptiPhase Hisafe 3 (PerkinElmer; catalog no.: 1200.437) scintillation liquid, and the radioactivity was measured using Wallac 1410 liquid scintillation counter. The results were normalized to total cell protein.

### Seahorse assay

The Agilent Seahorse XFe96 extracellular flux analyzer was used to analyze mitochondrial function. Reverse-transfected Huh-7 cells (2 × 10^4^ cells per well) were cultured in XF 96-well plate for 72 h. One hour prior to the assay, culture media were replaced with Seahorse XF base minimal DMEM (Agilent Technologies, Santa Clara, CA; catalog no.: 103334-100) supplemented with 1 mM sodium pyruvate (Sigma-Aldrich, Merck; catalog no.: S8636), 2 mM l-glutamine (Sigma-Aldrich, Merck; catalog no.: G7513), and 10 mM glucose (Sigma-Aldrich, Merck; catalog no.: G8769) for Mito stress assay. Modulators were injected at programmed intervals to achieve the final concentrations of 10 μM oligomycin (Sigma-Aldrich, Merck; catalog no.: O4876), 20 μM FCCP (Sigma-Aldrich, Merck; catalog no.: C2920), 10 μM rotenone (Sigma-Aldrich, Merck; catalog no.: R8875), and 10 μM antimycin A (Sigma-Aldrich, Merck; catalog no.: A8674). The oxygen consumption rate (OCR) values were further normalized to the number of cells present in each well, quantified by the Hoechst staining and counting by BioTek Cytation 5 Cell Imaging Multimode Reader (Agilent Technologies).

### Electron microscopy imaging and quantification

For electron microscopic analysis, the Huh-7 cells were grown on coverslips, thickness 0.13–0.16 mm. Post 72 h of transfection, the cells were fixed with 2% glutaraldehyde (electron microscopy [EM] grade) in 0.1 M sodium cacodylate buffer, pH 7.4, supplemented 2 mM calcium chloride, for 30 min, at room temperature. After washing with sodium cacodylate buffer, the cells were postfixed with 1% reduced osmium for 1 h, on ice, washed again with buffer, and dehydrated through increasing concentration of ethanol and acetone prior to infiltration into epoxy (TAAB 812, Aldermaston, UK). After polymerization of epoxy at +60° for 16 h, a pyramid was trimmed, and 60-nm-thick sections were cut and picked up on Pioloform-coated copper grids. The thin sections were poststained with uranyl acetate and lead citrate and imaged using a Hitachi HT7800 transmission electron microscope (Hitachi High-Technologies, Tokyo, Japan), operated at 100 kV, and equipped with a Rio9 CMOS-camera (AMETEK Gatan, Inc, Pleasanton, CA). About 11 cells from both control and GOLM1-silenced specimens were chosen using systematic random sampling, and all Golgi stacks in the chosen cell sections were imaged either by montaging and/or single images at 6,000× magnification. MIB software (38) was used to measure the lengths of the Golgi stacks with clear-cut profiles of cisterna, which represented 42.9% and 47.2% of the Golgi stacks in GOLM1-silenced and control cells, respectively.

### Proliferation analysis

The CellTiter 96® AQueous One Solution Cell Proliferation Assay kit (Promega, Madison, WI; catalog no.: G3582) was used to determine the cell proliferation according to the manufacturer’s protocol. Reverse-transfected Huh-7 cells (7.5 × 10^3^ cells per well) were transfected for 24, 48, and 72 h in a 96-well plate for the proliferation assay. The absorbance was measured at 490 nm using EnSpire Multimode plate reader from PerkinElmer.

### [^3^H] thymidine incorporation assay

Reverse-transfected Huh-7 cells were seeded on a 6-well plate (1 × 10^5^ cells per well) for 72 h. About 0.4 μCi/ml [^3^H] thymidine (Amersham, GE Healthcare; catalog no.: TRK686) was added and incubated for 4 h. The cells were washed three times with cold PBS and incubated with 5% trichloroacetic acid for 10 min, followed by 0.1 M NaOH treatment. After 10 min of incubation, the cells were scratched, and 3 ml of scintillation liquid (OptiPhase Hisafe 3) was added to the lysates. The radioactivity was measured using a Wallac 1410 liquid scintillation counter.

### Apoptosis sssay

Reverse-transfected Huh-7 cells (10 × 10^3^ cells per well) were seeded in opaque wall clear bottom 96-well plate (Corning Incorporated Life Sciences, Kennebunk, ME; catalog no.: 3610). Post 24 h of transfection, 2× detection reagent from RealTime-Glo™ Annexin V Apoptosis and Necrosis Assay Kit (Promega; catalog no.: JA1011) was added on the cells. Luminescence (relative light unit) measures were taken at different time points in an EnSpire Multimode plate reader (PerkinElmer).

### Flow cytometry cell cycle analysis

Reverse-transfected Huh-7 cells (5 × 10^5^ cells per well) were cultured for 72 h in a 6-well plate. The cells were then trypsinized and centrifuged. The cells were washed for three times with PBS, and the pellet was suspended in 500 μl of propidium iodide solution (0.05 mg/ml propidium iodide, 3.8 μM sodium citrate, and 0.1% Triton X-100 in PBS) and incubated for 15 min at room temperature. The samples were then run with BD Accuri C6 flow cytometer with FL2 detector, and the results were analyzed with FlowJo_v10.8.0 software (BD Bioscience) using the univariate Dean-Jett-Fox cell cycle model. The flow cytometry analysis was performed at the HiLIFE Biomedicum Flow Cytometry Unit, University of Helsinki.

### Statistics

The Mann-Whitney *U* test was used to compare the differences between two groups. The data are depicted as mean ± SD, and *P* value of <0.05 was considered as statistically significant. For lipidomic analysis, multiple Mann-Whitney *U* tests were used. Differences in GOLM1 expression in the GSE datasets were compared using the one-way ANOVA. An adjusted *P* value of <0.05 was considered as statistically significant. GraphPad Prism 9.3.1 (GraphPad Software, Inc) or R 4.0.3 was used for the statistical analyses.

## Results

### *GOLM1* expression is increased in HCC with different etiologies

**Fig. 1:**
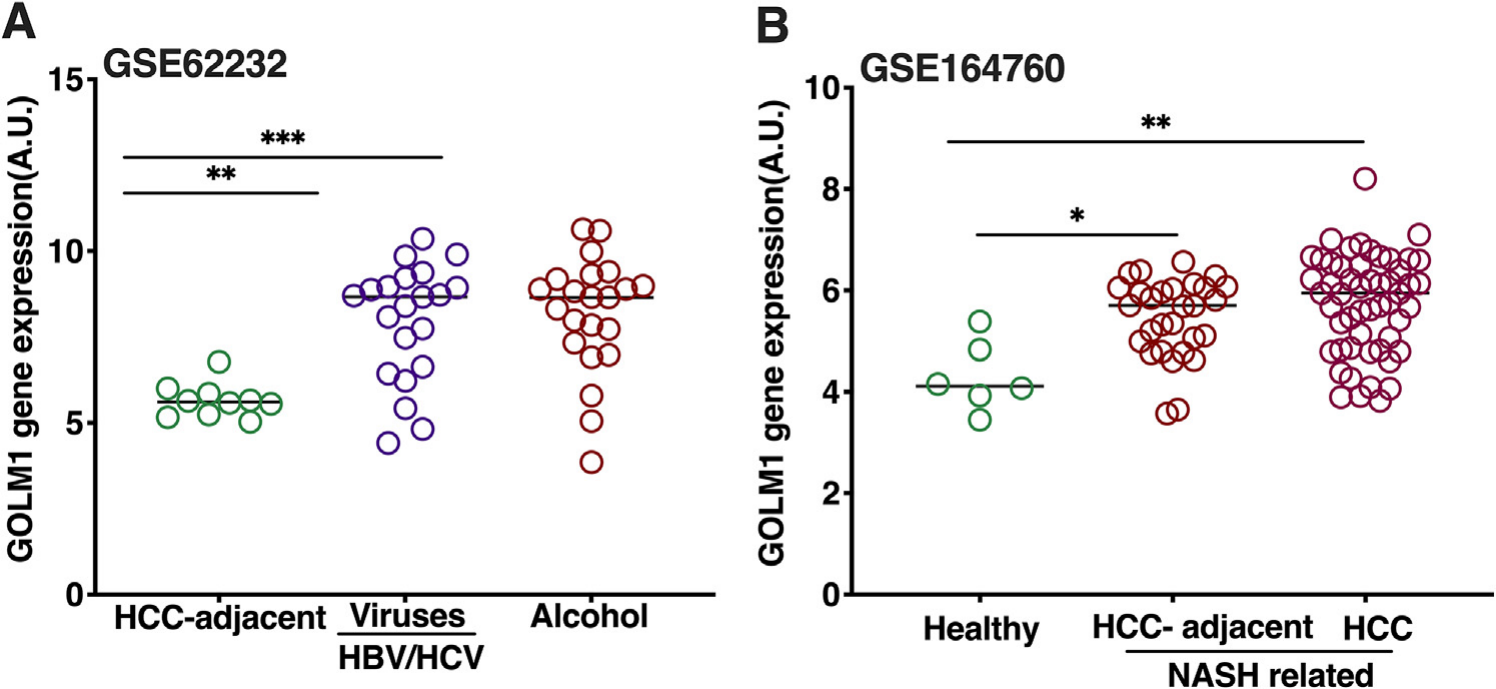
*GOLM1* gene expression in HCC cohorts with different etiologies. A: *GOLM1* expression in tumor adjacent normal samples and liver tumors caused by alcohol and HBV or HCV (data obtained from GSE62232 dataset). B: *GOLM1* expression in HCC caused by NAFLD (data from dataset GSE164760), NASH-HCC tumors, and NASH-HCC adjacent samples compared with healthy controls. Data are represented as mean ± SD. ∗∗∗*P_adj_* < 0.001, ∗∗*P_adj_* < 0.01, and ∗*P_adj_* < 0.05.

**Fig. 2:**
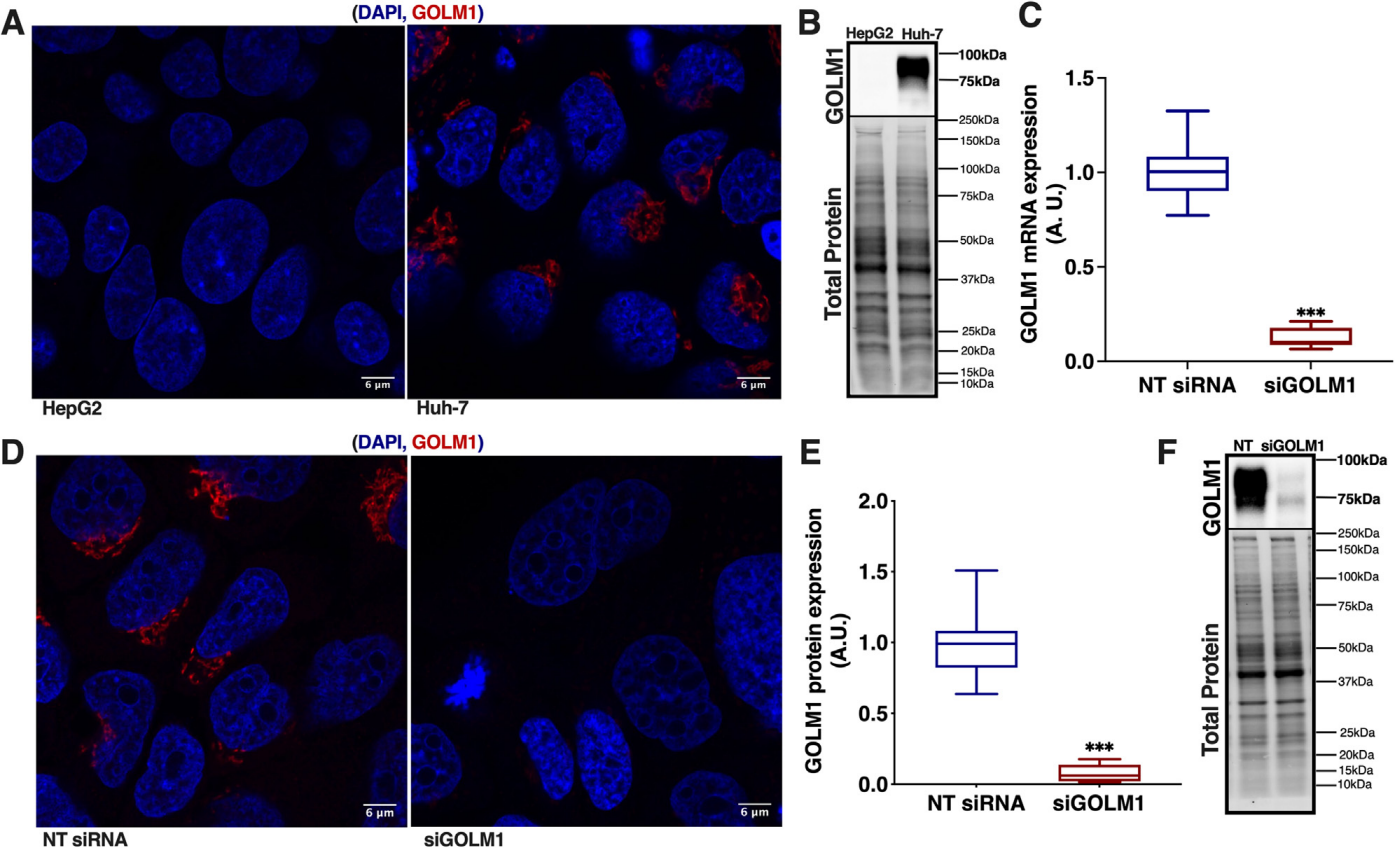
GOLM1 expression in different HCC models. A: Anti-GOLM1 staining in HepG2 and Huh-7 cells for endogenous GOLM1 expression. The scale bar represents 6 μm. B: Western blot of GOLM1 in HepG2 and Huh-7 cells. C: mRNA expression of *GOLM1* in Huh-7 cells subjected to its knockdown. D: Anti-GOLM1 staining in Huh-7 cells subjected to GOLM1 silencing. The scale bar represents 6 μm. E: Quantification of GOLM1 protein expression in Huh-7 cells subjected to GOLM1 knockdown. F: Western blot of GOLM1 in NT (nontargeting) siRNA and siGOLM1 Huh-7 cells. Data are represented as mean ± SD, all experiments are repeated at least three times with multiple replicates in each set. ∗∗∗*P* < 0.001. The representative full Western blot is available in supplemental data section.

NAFLD, hepatitis viruses, alcohol and toxin exposure are some of the causes associated with the development of HCC. Therefore, the expression of *GOLM1* in HCC with different etiologies was analyzed using the data from the publicly available datasets GSE62232 and GSE164760. *GOLM1* mRNA expression was elevated in HCC related with hepatitis viruses, high alcohol consumption, and NASH (Fig. 1A, B). To study the effect of GOLM1 depletion on lipid metabolism in HCC cell lines, the Huh-7 cell line was selected because of its higher expression of GOLM1 compared with HepG2 cells (Fig. 2A, B). GOLM1 expression was silenced in Huh-7 cells for 72 h with siRNA, resulting in significant reductions at both mRNA and protein levels, by 88% and 90%, respectively (Fig. 2C–F).

### GOLM1 depletion alters sphingolipids along with other lipid classes

**Fig. 3:**
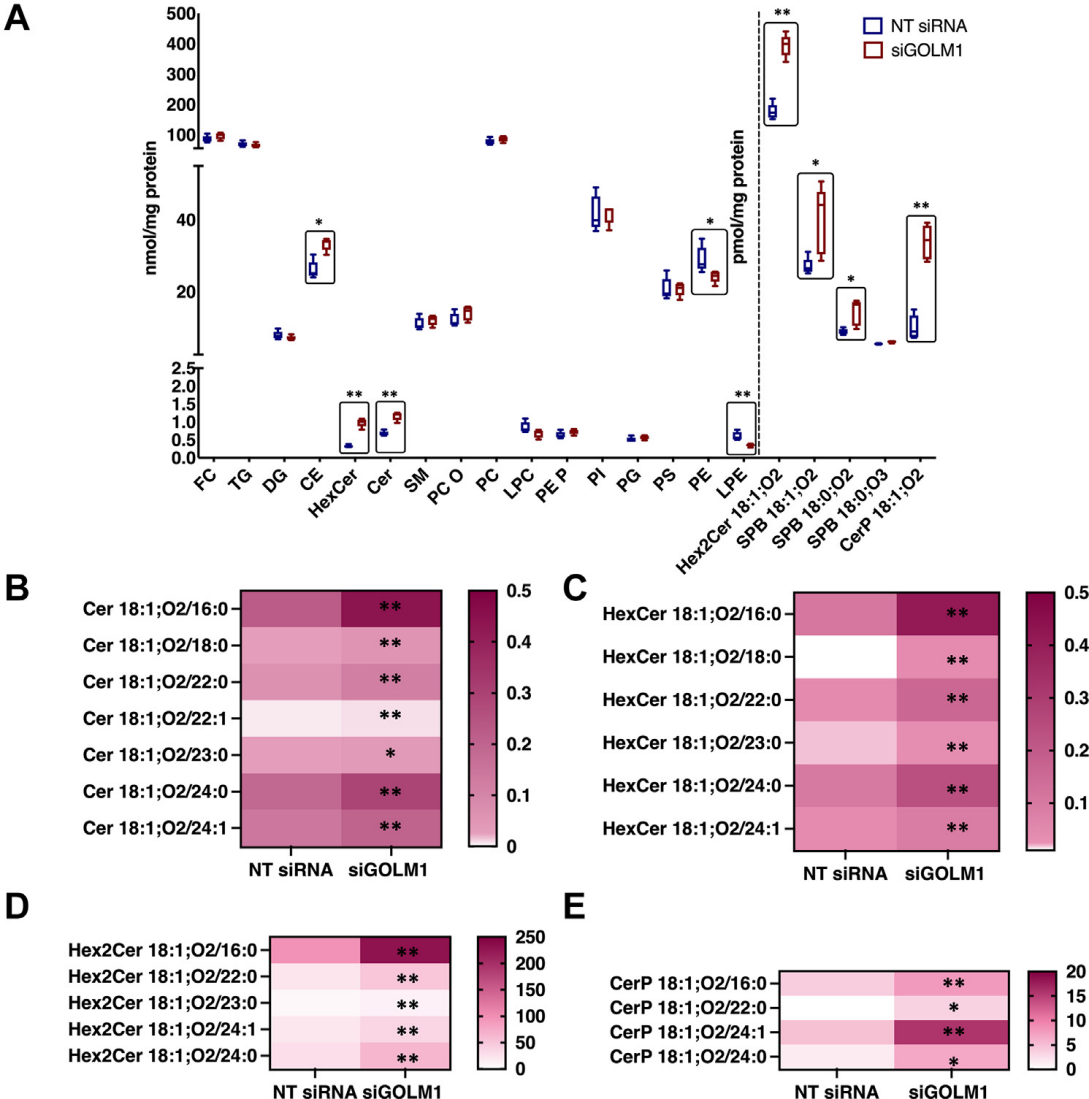
Lipidome profile of GOLM1-silenced and control Huh-7 cells. A: Total lipid classes analyzed in GOLM1-silenced and control Huh-7 cells. The boxes highlight the significantly altered lipid classes and species. B: Concentrations of each individual species of ceramides in control (NT siRNA) and GOLM1-silenced (siGOLM1) cells. C: Individual hexosylceramide species. D: Individual dihexosylceramide species. E: Individual ceramide phosphate species. Data are represented as lipid concentrations, mean ± SD (N = 5). ∗∗*P* < 0.01 and ∗*P* < 0.05.

**Fig. 4:**
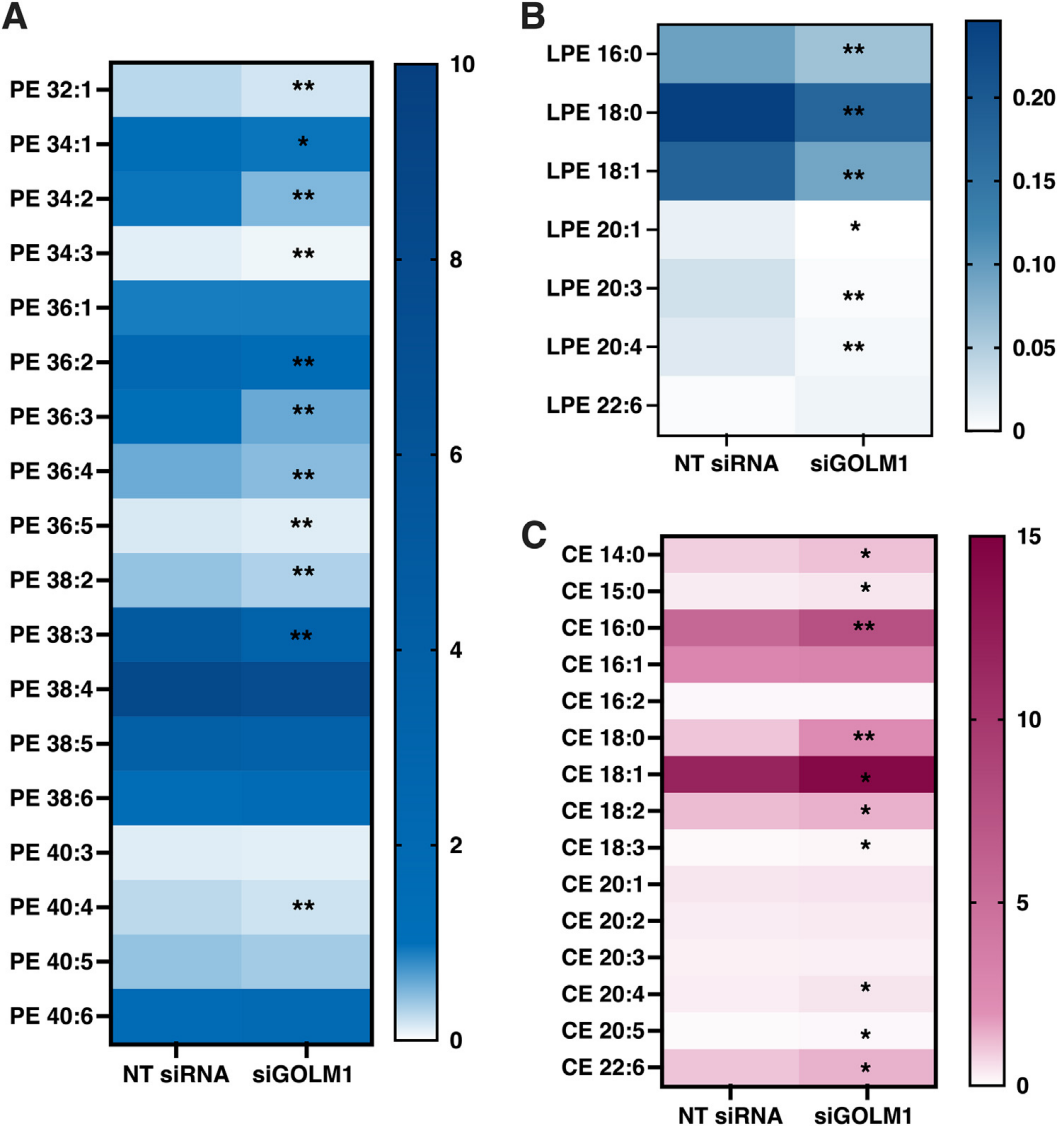
The alterations of membrane phospholipid and neutral lipid species concentrations upon GOLM1 knockdown. A: Individual phosphatidylethanolamine species. B: Individual lysophosphatidylethanolamine species. C: Individual CE species. Data are represented as lipid concentrations, mean ± SD (N = 5). ∗∗*P* < 0.01 and ∗*P* < 0.05.

To analyze the effect of GOLM1 depletion on HCC lipid metabolism, GOLM1-depleted Huh-7 cells were subjected to lipidomic analysis by MS. A drastic accumulation of Cer, HexCer, and Hex2Cer (most likely lactosyl ceramide), sphingosine (SPB 18:1;O2), sphinganine (SPB 18:0;O2), and CerP along with CE was observed in the GOLM1-depleted cells as compared with controls, whereas a significant reduction was seen in membrane phospholipids such as LPE and PE (Fig. 3A). Individual species of different sphingolipid classes like Cer 18:1;O2/16:0, 18:1;O2/18:0, 18:1;O2/22:0, 18:1;O2/22:1, 18:1;O2/24:0, 18:1;O2/24:1, 18:1;O2/23:0 (Fig. 3B); HexCer 18:1;O2/16:0, 18:1;O2/18:0, 18:1;O2/22:0, 18:1;O2/23:0, 18:1;O2/24:0, 18:1;O2/24:1 (Fig. 3C); Hex2Cer 18:1;O2/16:0, 18:1;O2/22:0, 18:1;O2/23:0, 18:1;O2/24:1, 18:1;O2/24:0 (Fig. 3D); CerP 18:1;O2/16:0, 18:1;O2/24:1, 18:1;O2/22:0, 18:1;O2/24:0 (Fig. 3E) were significantly higher in the GOLM1 knockdown cells. In addition, an increase in sphingolipid synthesis was also observed (supplemental Fig. S1) in GOLM1 knockdown cells. A number of membrane phospholipid species such as PE 32:1, 34:1, 34:2, 34:3, 36:2, 36:3, 36:4, 36:5, 38:2, 38:3, 40:4 and LPE 16:0, 18:0, 18:1, 20:3, 20:4, 20:1 were significantly downregulated in the knockdown cells (Fig. 4A, B).

### GOLM1 silencing increases CE accumulation

**Fig. 5:**
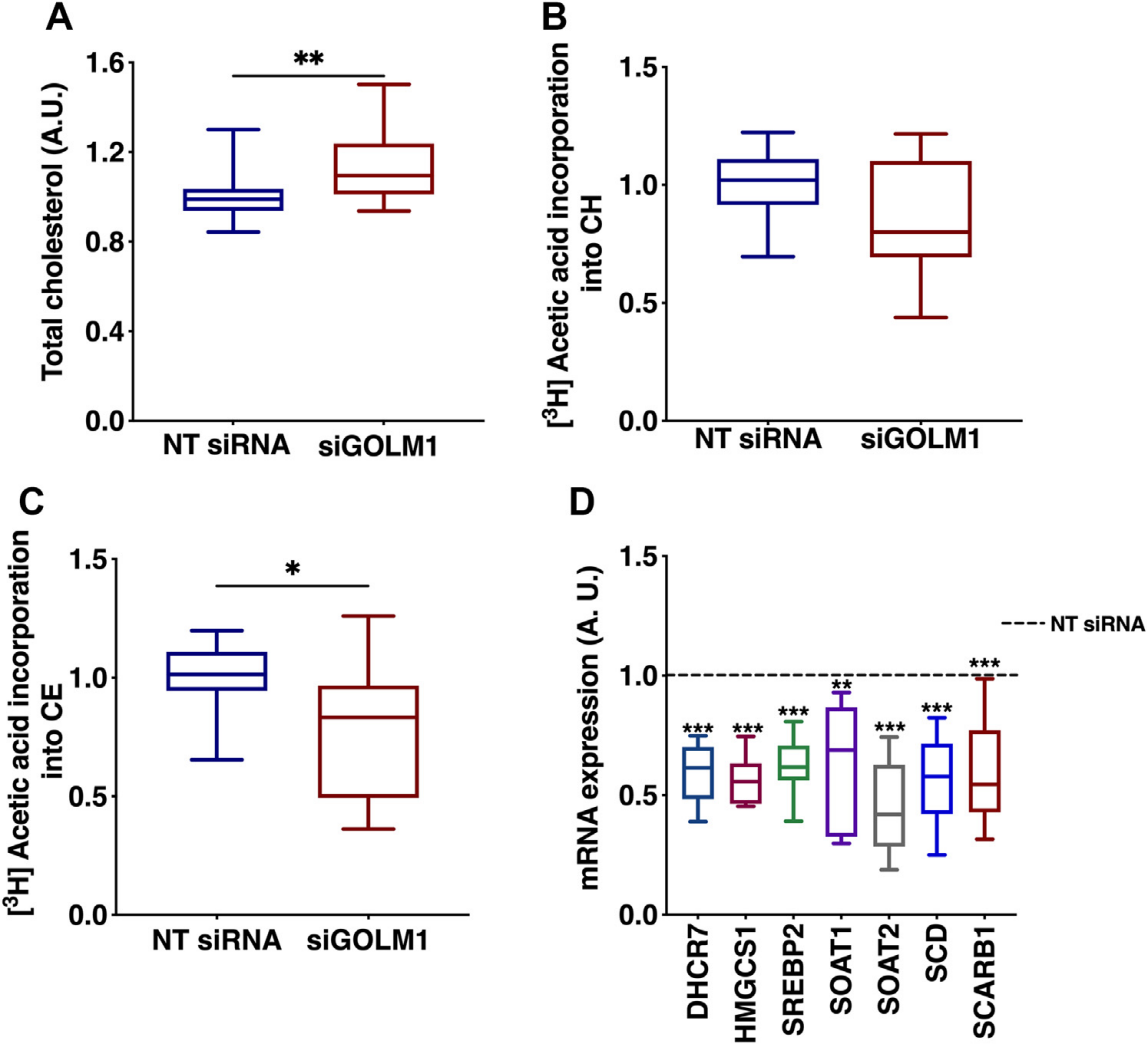
CE accumulation in GOLM1-silenced Huh-7 cells. A: Enzymatic assay to measure total cholesterol content in GOLM1 knockdown cells. B: [^3^H] acetic acid incorporation (de novo synthesis) into cholesterol (CH). C: [^3^H] acetic acid incorporation (de novo synthesis) into CEs. D: mRNA expression of CH and CE synthesis genes (dashed [---] line represents NT siRNA). Data are represented as mean ± SD, all experiments were repeated at least three times with multiple replicates in each set. ∗∗∗*P* < 0.001, ∗∗*P* < 0.01, and ∗*P* < 0.05.

Since an increase in total CE was observed in the GOLM1-depleted cells, changes in CE species were analyzed. Significant increases in the levels of CE species 16:0, 18:0, 14:0, 15:0, 18:1, 18:2, 18:3, 20:4, 20:5, and 22:6 were observed in the GOLM1 knockdown cells (Fig. 4C). Consistent with this observation, total cholesterol assayed by an enzymatic method was also increased (Fig. 5A). In order to see if the elevation of CE is due to an increase in cholesterol synthesis, de novo lipogenesis assays were performed by labeling the cells with [^3^H] acetic acid. There was no increase in the synthesis of CE or free cholesterol, rather a tendency of decrease in cholesterol and a significant decrease in CE synthesis was observed (Fig. 5B, C). Consistent with this result, the mRNA expression levels of cholesterol or CE synthetic genes (7-dehydrocholesterol reductase [*DHCR7*], 3-hydroxy-3-methylglutaryl-CoA synthase 1 [*HMGCS1*], sterol O-acyltransferase 1 [*SOAT 1*], sterol O-acyltransferase 2 [*SOAT2*], sterol regulatory element binding transcription factor 2 [*SREBP2*], stearoyl-coenzyme A desaturase [*SCD*], scavenger receptor class B member 1 [*SCARB1*]) were decreased (Fig. 5D).

### GOLM1 knockdown reduces mitochondrial OCR

**Fig. 6:**
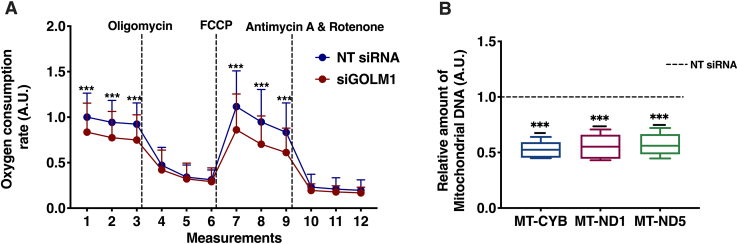
Mitochondrial stress test and mitochondrial DNA (MtDNA) content in GOLM1-silenced Huh-7 cells. A: OCR was measured in real time for GOLM1 knockdown and control Huh-7 cells. B: MtDNA content in GOLM1-silenced and control Huh-7 cells by using real-time PCR (dashed [---] line represents NT SIRNA). Data are represented as mean ± SD, from three experiments each with multiple replicates. ∗∗∗*P* < 0.001.

Higher ceramides, glucosyl ceramide (GlcCer), and lactosylceramide (LacCer) in liver cells are known to adversely affect the mitochondrial function (39, 40). Moreover, changes in mitochondrial PE content are known to affect mitochondrial stability and function (41). As we observed an increase in Cer, HexCer, and Hex2cer (LacCer) and a decrease in PE, mitochondrial OCR was analyzed in GOLM1-silenced and control cells. A significant reduction in both basal OCR and the mitochondrial maximal respiration were observed in GOLM1-silenced cells compared with control cells (Fig. 6A). To study whether PE supplementation could rescue the mitochondrial function, these cells were treated with and without PE-containing vesicles and methyl-α-cyclodextrin, which transfers PE to the cells, followed by measurement of mitochondrial respiration. Significant increase in mitochondrial respiration (OCR) was detected in both PE-treated control and GOLM1 knockdown cells (supplemental Fig. S2). A rescue effect on respiration in the GOLM1-silenced cells was seen compared with untreated GOLM1-silenced cells. However, the increase in the respiration in PE-treated GOLM1 knockdown cells did not reach the level in PE-treated control cells.

Furthermore, a decrease in mitochondrial DNA content (mitochondrially encoded cytochrome B [MT-CYB], mitochondrially encoded NADH:ubiquinone oxidoreductase core subunit 1 [MT-ND1], mitochondrially encoded NADH:ubiquinone oxidoreductase core subunit 5 [MT-ND5]) was observed in GOLM1 knockdown cells as compared with controls (Fig. 6B).

### GOLM1 reduction leads to distortion of Golgi structure

**Fig. 7:**
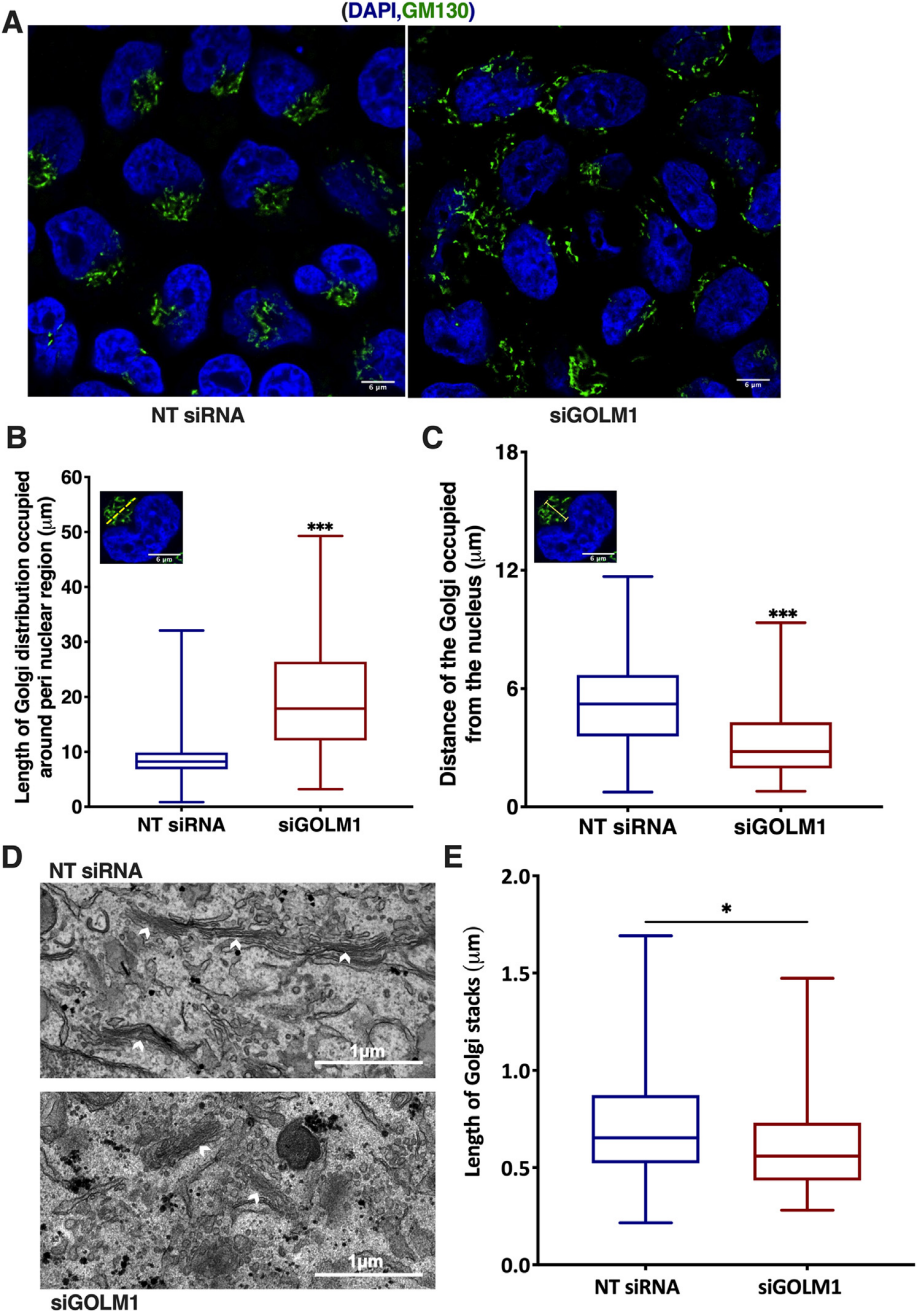
Golgi membrane disintegration when GOLM1 is knocked down in Huh-7 cells. A: Anti-Golgin subfamily A member 2 (GM130) staining in GOLM1-silenced and control Huh-7 cells. B: Quantification of Golgi distribution around perinuclear region in anti-GM130-stained specimens. C: Quantification of distance of the Golgi from the nucleus in anti-GM130-stained specimens (number of cells = 104–115). The scale bar represents 6 μm. Same single-cell image is inserted in graphs B and C, and it is only for illustrating the principle of two different measurements (yellow dotted line in B inset image indicates the perinuclear Golgi distribution measurement, and yellow arrow line in C indicates Golgi length measurement from the nucleus). D: Transmission electron micrographs showing Golgi stacks in GOLM1-silenced and control Huh-7 cells, the arrowheads indicate the clear-cut stack profiles subjected to quantitation. The scale bars represent 1 μm. E: Quantification of Golgi stack lengths in control and GOLM1 knockdown cells (68 and 63 stacks from 11 cells, respectively). ∗∗∗*P* < 0.001 and ∗*P* < 0.05.

Since GOLM1 is a Golgi localized protein and an aberrant increase in HexCer was observed in GOLM1 knockdown cells, further efforts were made to study whether GOLM1 silencing affects Golgi structure and morphology. Control and GOLM1-silenced cells were stained for GM130, and its distribution was measured. Scattering of the Golgi stacks was observed in GOLM1-depleted cells (Fig. 7A). In GOLM1-depleted cells, the Golgi structures were not concentrated on one side of the nucleus as in the control cells, but instead, scattered distribution of the Golgi structures around the perinuclear region was observed (Fig. 7B, C). Consistently, the scattered distribution of Golgi stacks was observed with thin section transmission electron microscopy. In addition, a quantitative EM analysis demonstrated a reduction in the length of the Golgi stacks (Fig. 7D, E). Together, the light microscopy and EM data suggest a breakdown of the Golgi ribbon followed by a shortening and redistribution of Golgi stacks around the nucleus upon GOLM1 silencing.

### Depletion in GOLM1 affects cell growth and apoptosis

**Fig. 8:**
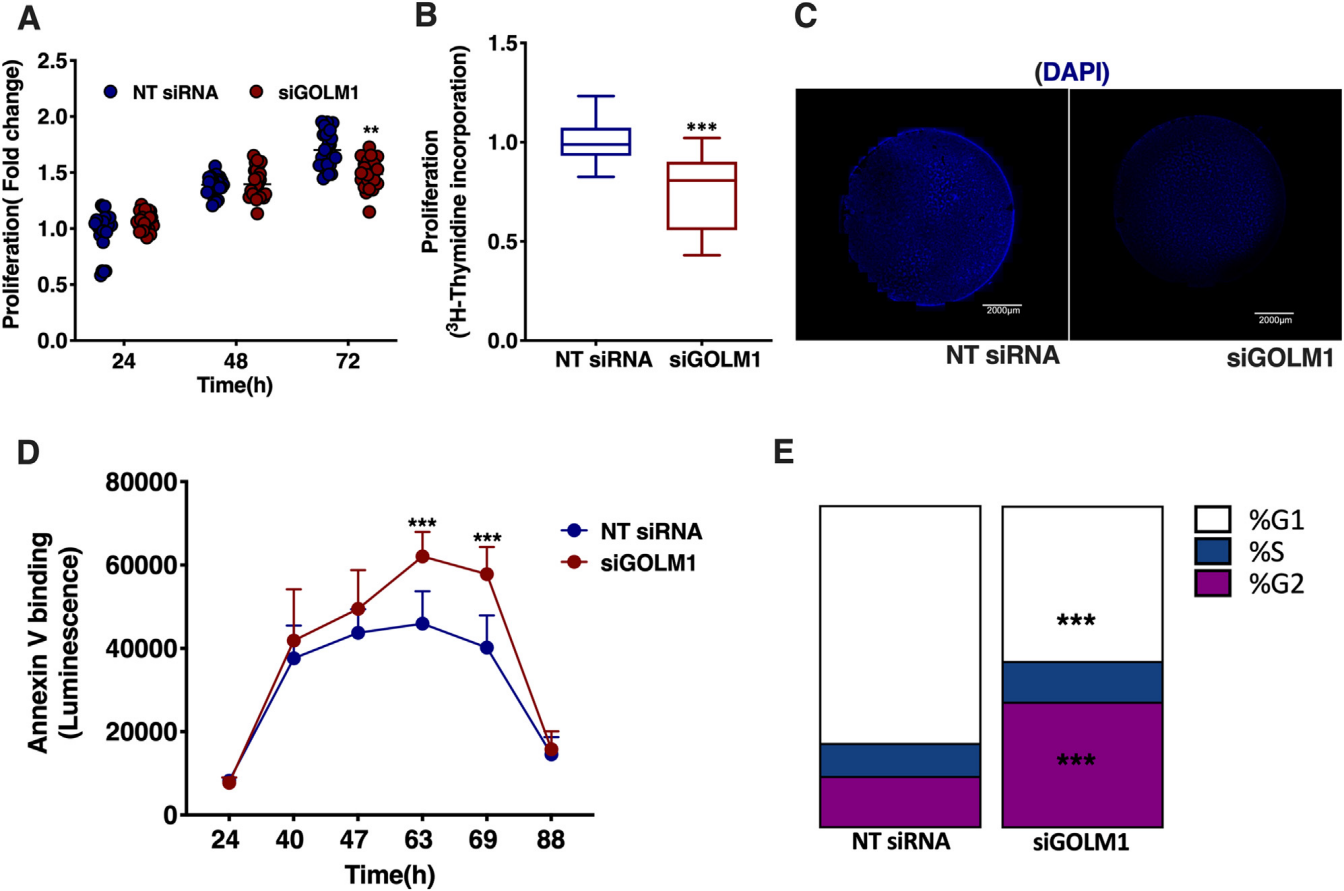
Silencing GOLM1 expression in Huh-7 cells modulates cell proliferation, apoptosis, and cell cycle. A: CellTiter 96® AQueous One Solution Cell Proliferation Assay (MTT) to measure cell proliferation in GOLM1-silenced Huh-7 cells at 24, 48, and 72 h. B: [^3^H] thymidine incorporation assay to measure proliferation. C: DAPI nuclear staining of GOLM1-depleted and control Huh-7 cells grown on coverslips. Coverslip slides were scanned using 3D HISTECH Panoramic 250 Flash III, and snapshots were taken at 0.8× magnification using CaseViewer, version 2.2. The scale bar represents 2,000 μm. D: RealTime-GloTM Annexin V Apoptosis Assay to measure apoptosis from 24 to 88 h in GOLM1-silenced cells. E: Cell cycle analysis of GOLM1 knockdown and control cells. Data are represented as mean ± SD, all experiments were repeated at least three times with multiple replicates in each set. ∗∗∗*P* < 0.001 and ∗∗*P* < 0.01. DAPI, 4′,6-diamidino-2-phenylindole.

The effects of GOLM1 silencing on cell proliferation, cell cycle, and apoptosis were further analyzed. A modest but significant reduction in proliferation was observed at 72 h in GOLM1-silenced cells by one-step 3-(4,5-dimethylthiazol-2-yl)-2,5-diphenyl tetrazolium bromide proliferation assay (Fig. 8A). However, in a cell proliferation assay employing [^3^H] thymidine labeling, 22.5% reduction was observed at 72 h of GOLM1 silencing (Fig. 8B). Furthermore, nuclear staining with 4′,6-diamidino-2-phenylindole also showed a reduction in the total intensity coverslips harboring GOLM1 knockdown and control cells, consistent with a reduction in cell proliferation (Fig. 8C). Furthermore, an increase in apoptosis was seen between 48 and 72 h in GOLM1 knockdown cells by using annexin V apoptosis assay (Fig. 8D). Cell cycle analysis revealed an enrichment of G2 and S phase cell populations in the GOLM1-silenced cells as compared with control cells. Correspondingly, G1 phase was reduced in the GOLM1-depleted cells (equal numbers of cells were taken for the analysis from both control and GOLM1 knockdown preparations) (Fig. 8E).

### GOLM1 knockdown affects the expression of sphingolipid metabolism genes and Golgi proteins

**Fig. 9:**
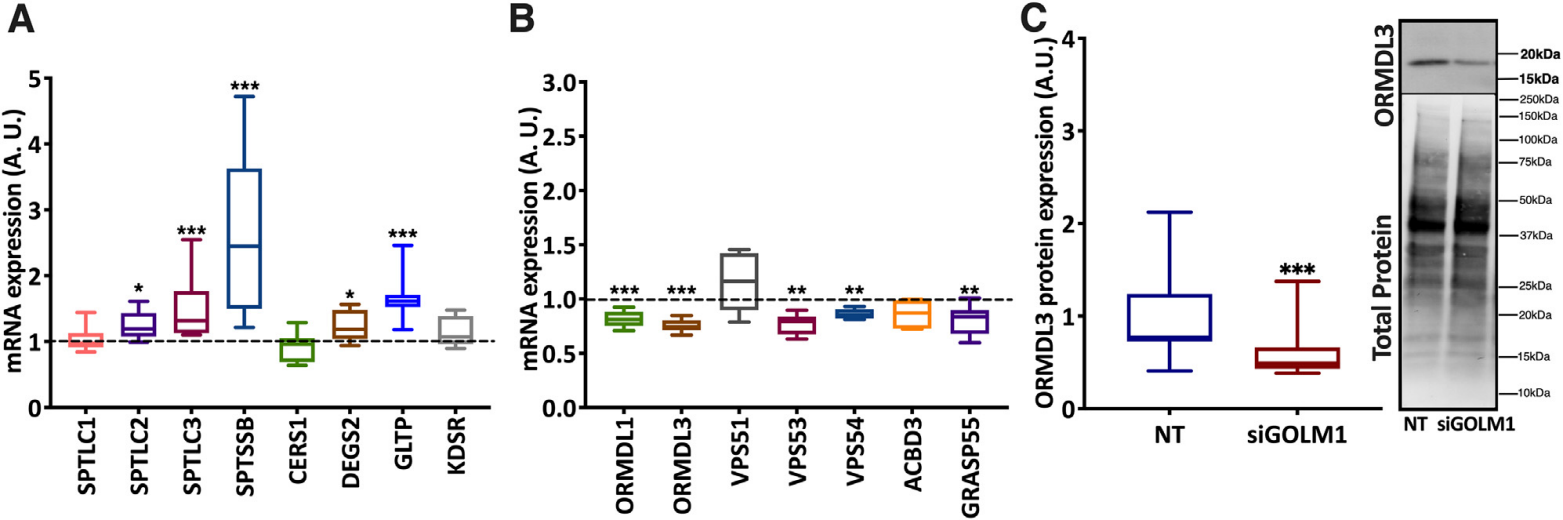
GOLM1 depletion alters the expression of genes involved in ceramide synthesis or encoding Golgi proteins. A: mRNA expression of genes involved in sphingolipid metabolism. B: mRNA expression of sphingolipid biosynthesis regulators. C: Western blot of ORMDL3 in NT siRNA and siGOLM1-transfected Huh-7 cells (dashed [---] line represents NT siRNA). Data are represented as mean ± SD, all experiments were repeated at least three times with multiple replicates in each set. ∗∗∗*P* < 0.001, ∗∗*P* < 0.01, and ∗*P* < 0.05. A representative full Western blot is available in supplemental data section.

Since ceramide and CE accumulations as well as Golgi structural alterations were observed upon GOLM1 silencing, mRNA expression of ceramide synthetic genes and Golgi proteins was analyzed. A significant increase in mRNA expression of genes involved in sphingolipid synthesis such as serine palmitoyltransferase, long chain base subunit 2 (*SPTLC2*), serine palmitoyltransferase, long chain base subunit 2 (*SPTLC3*), serine palmitoyltransferase small subunit B (*SPTSSB*), delta 4-desaturase, sphingolipid 2 (*DEGS2*), and glycolipid transfer protein (*GLTP*) were observed (Fig. 9A). Furthermore, a significant reduction in ORMDL sphingolipid biosynthesis regulator 1 (*ORMDL1*) and ORMDL sphingolipid biosynthesis regulator 3 (*ORMDL3*), negative regulators of ceramide synthesis, was seen (Fig. 9B), and reduction of ORMDL3 was also verified at the protein level (Fig. 9C). In addition, in the GOLM1 knockdown cells, *VPS53* (vacuolar protein sorting 53) and *VPS54*, GARP (Golgi-associated retrograde protein) complex components were mildly but significantly downregulated (Fig. 9B). GARP complex dysfunction is known to induce sterol and sphingolipid accumulation (42). Depletion of acyl-CoA binding domain containing 3 (ACBD3), also known as GOLPH1, leads to Golgi fragmentation and alters glycosphingolipid (GSL) metabolism (43). A mild reduction of *ACBD3* mRNA expression was observed in GOLM1-silenced cells (Fig. 9B). Furthermore, expression of Golgi reassembly-stacking protein 2, 55 kDa (*GRASP55*) encoding another Golgi protein involved in GSL metabolism (44), was downregulated in the GOLM1 knockdown cells (Fig. 9B).

## Discussion

GOLM1/GP73 is known to be involved in the pathogenesis of many cancers, and its expression is elevated in hepatocytes also under other disease conditions. Importantly, *GOLM1/GP73* expression is elevated in HCC (5, 7, 8, 45, 46). Many studies suggest that *GOLM1* expression is elevated mainly in viral-mediated HCC as its expression is known to be induced upon viral infections including SARS-CoV2 (2, 5, 47). However, a very recent study demonstrated that its expression is also elevated in nonobese NAFLD (7). In the present study, using data from publicly available databases GSE62232 and GSE164760, expression of *GOLM1* in HCC with different etiologies was analyzed. The data (Fig. 1A, B) show that irrespective of the etiology, *GOLM1* expression is significantly elevated in HCC. Many studies have shown that targeting GOLM1 affects cellular growth and metabolism (13, 45, 48). However, so far, very little data are available on how GOLM1 affects the lipid profile and metabolism of HCC cells. Therefore, in this study, extensive lipid profiling of GOLM1-silenced and control HCC cells was carried out.

Lipidomic profiling of GOLM1 knockdown cells showed alterations in many lipid classes. Drastic increases of several sphingolipids were observed in GOLM1 knockdown cells, especially in Cer, HexCer, Hex2Cer, sphingosine, sphinganine, and CerP. The elevated concentrations of ceramides and their precursor sphinganine indicate an enhanced synthesis of ceramides and its higher forms in GOLM1 knockdown cells. In agreement with these results, the mRNAs of ceramide synthetic genes *SPTLC2*, *SPTLC3*, *SPTSSB*, *DEGS2*, and *GLTP* were increased in the knockdown cells. Furthermore, an assay of sphingolipid synthesis by [^3^H] serine labeling also showed a mild increase in the knockdown cells (supplemental Fig. S1). Consistent with these results, *ORMDL1* and *ORMDL3*, negative regulators of de novo ceramide synthesis, were downregulated in the GOLM1 knockdown cells. Reduction of ORMDLs is known to increase sphingosine, sphinganine, Cer, HexCer, and LacCer (49). Double knockdown of ORMDL1 and ORMDL3 or ORMDL2 and ORMDL3 has a synergistic effect on the accumulation of sphingosines, sphinganines, and ceramides (50, 51). These observations suggest that ORMDLs might have an important role in the GOLM1-mediated sphingolipid alterations. The increase in both sphingosine and sphinganine in GOLM1 knockdown cells implies that the accumulating ceramide is possibly generated via both the synthetic and the salvage pathways. However, the increased SM seen upon ORMDL3 knockdown was not observed in the GOLM1 knockdown cells. SM total levels were not altered in GOLM1 knockdown cells even though there was a small increase observed in SM synthesis in these cells (supplemental Fig. S1).

Depletions of a number of other Golgi proteins, such as ACBD3, Golgi phosphoprotein 3 (GOLPH3), GRASP55, and pleckstrin homology domain containing A8 (PLEKHA8-FAPP2), are known to affect sphingolipid and GSL metabolism (43, 44, 52, 53). Depletion of GRASP55/GOLPH6, which is involved in the compartmentalization of GSL synthetic enzymes in Golgi, also resulted in aberrant increase of GlcCer, LacCer, and globosides (44). *ACDB3* and *GRASP55* expression were mildly reduced in the GOLM1 knockdown cells, which might also contribute to the altered GSL levels. Knockdown of ACBD3/GOLPH1 leads to accumulation of GlcCer, SM, and sphingosine mainly because of a defect in GlcCer transport to *trans* Golgi network via FAPP2, which in turn results in defective synthesis of higher GSLs, such as LacCer, monosialodihexosylganglioside (GM3), and globotriaosylceramide (GB3) (43). A mild reduction in *ACBD3* expression, however, did not result in the reduction of Hex2Cer (LacCer), rather an increase was noted suggesting that GlcCer transport to the *trans* Golgi network by FAPP2 may not be altered in these cells. Taken together, these data suggest that GOLM1 potentially plays a role in GSL metabolism similar to the other GOLPHs, possibly by regulating the intra-Golgi transport. The GARP complexes are involved in the retrograde transport from endosomes to Golgi. Deletion of a GARP complex component VPS53 caused an increase in long-chain bases like sphingosine, sphinganine, Cer, and HexCer. In addition, VPS53 and VPS54 mutants exhibited growth defects in yeast (42). VPS53 silencing also induces sterol ester accumulation in yeast without an increase in cholesterol synthesis. Similar to this observation, GOLM1 knockdown cells also exhibited increased accumulation of CEs without a significant increase in cholesterol synthesis. A mild but significant reduction was observed in VPS53 and VPS54 expression in GOLM1 knockdown cells. Taken together, these data suggest that a functional association of GOLM1 with the GARP complex might exist (42), contributing to increased sphingolipids and CEs.

In addition to alterations in sphingolipids and CEs, GOLM1 knockdown cells also displayed a reduction in PE and LPE. This might be due to a defect in mitochondrial function in these cells (54). On the other hand, PE reduction can also lead to mitochondrial dysfunction (55). Therefore, mitochondrial respiration (OCR) was analyzed in the GOLM1 knockdown cells revealing a decrease in OCR as compared with the controls. We find it likely that the increased Cer and reduced PE concentrations may be one of the reasons for this defect. Alterations in PE levels do affect the mitochondrial function (41). PE supplementation in control and GOLM1 knocked down cells exhibited an increase in mitochondrial respiration, further confirming the importance of PE for mitochondrial respiration. PE supplementation rescued the defect in mitochondrial respiration in GOLM1 knockdown cells. However, the respiration rate in PE-supplemented GOLM1-silenced cells did not increase up to the levels in PE-supplemented control cells. This might be due to the reduced mitochondrial content or increased sphingolipid levels observed in the knockdown cells after 72 h of silencing. Thus, the reduced levels of PE in GOLM1-silenced cells might contribute to the defect in mitochondrial respiration in these cells. In addition, total mitochondrial content was also seen reduced in these cells. Similarly, defects in mitochondrial function were also detected upon ORMDL3 knockdown (56).

A tight connection exists between Golgi morphology and sphingolipid metabolism. The Golgi resident proteins, GRASPs, are shown to maintain Golgi structure and function (57). Importantly, these proteins are also involved in sphingolipid metabolism similar to GOLM1. Since an aberrant accumulation of sphingolipids is seen upon GOLM1 knockdown, we considered it possible that some Golgi structural defect may also exist in these cells. Further experiments showed that the GOLM1 knockdown cells displayed a scattered pattern of GM130 staining (Golgi marker) as compared with more intact concentrated stacks in control cells. EM imaging verified a defect in Golgi stack length. The defect in Golgi structure in these cells might be partially mediated through the reduction of ACBD3 and GRASP55 (43, 57). To understand the relationship between Golgi structural integrity and altered sphingolipid metabolism in the GOLM1 knockdown cells, more studies are warranted. An enrichment of G2 phase cells was seen when GOLM1 was silenced, whereas G1 phase cells were decreased as compared with control cells, indicating a possible delayed entry into mitosis. The observed enrichment of G2 cell population can be a consequence from enhanced ceramide accumulation (58). GOLM1 is shown to enhance proliferation of cancer cells (59). In this study, cell cycle arrest, decreased proliferation, viability, and increased early apoptosis were observed in the GOLM1 knockdown cells, in agreement with the reduced mitochondrial function and ceramide accumulation in these cells.

In conclusion, GOLM1 depletion in hepatocarcinoma cells resulted in aberrant accumulation of sphingolipids and CEs, possibly because of a defect in Golgi structure and function in retrograde trafficking. These changes in lipids were associated with reduced mitochondrial function and cell proliferation indicating putative future value of GOLM1 as a therapy target for cancers.

## Data availability

All data used in this study are presented as main figures or supplemental figures and tables.

## Supplemental data

This article contains supplemental data.

## Conflict of interest

The authors declare that they have no conflicts of interest with the contents of this article.
