# Supplementary material for: GOLM1 depletion modifies cellular sphingolipid metabolism and adversely affects cell growth
Source: J Lipid Res. 2022 Aug 7;63(9):100259. doi: 10.1016/j.jlr.2022.100259 (PMC9475319; doi:10.1016/j.jlr.2022.100259)

**Supplementary methods:**

***Analysis of sphingolipid synthesis by [^3^H]serine labelling***

GOLM1 silenced and control cells were grown in 6-well plate and labelled with 1μCi/well [^3^H]serine (PerkinElmer, Waltham, MA, USA, NET248250UC) for 4 hours post 72 hours transfection, and the total lipids were extracted and subjected to alkaline hydrolysis of phospholipids. The hydrolyzed samples were resolved using chloroform:methanol:acetic acid:water (60:40:4:1, vol/vol) with lipid standards for sphinganine, sphingomyelin, ceramide and hexosyl ceramide. Radioactivity was measured using a Wallac 1410 liquid scintillation counter with Hisafe 3 scintillation liquid. The results were normalized to total cell protein. Due to resolution issues, the radioactive measurements of hexosyl ceramides, ceramides and sphinganine were combined.

***PE loading and mitochondrial respiration analysis***

GOLM1 knockdown and control cells (post 72 hours transfection) were treated with and without PE (Avanti, Sigma-Aldrich, Merck, 850725P) vesicles for 4 hours and mitochondrial respiration (OCR) was measured by Seahorse XF96 analyzer as detailed in the Materials and Methods section ‘Seahorse assay’. PE vesicles were made in the ratio 20:1-20:1-PC (65 mol%) : 18:1-18:1-PE (30 mol%) : Cardiolipin (5 mol%). Lipids were dried and dissolved in serum free medium, 20 mM HEPES, pH 7.4, and sonicated 3 times for 2 minutes with 30 seconds intervals and large particles were removed by centrifugation at 5000 x g for 5 minutes. The lipid vesicle supernatant was collected and mixed with 8 mM methyl-α-cyclodextrin solution. For control, serum free medium without lipids was sonicated as mentioned above and the same concentration of methyl-α-cyclodextrin was added. One-way ANOVA was used to compare the differences between the groups.

**Supplementary Table 1. Primers employed for the qPCR analyses.**

| **Gene** | **Primer sequence** |
| --- | --- |
| ACBD3 F | CTTGTGGCACTGCATAAGCAA |
| ACBD3 R | TCTATTTTGTGGGACGCAACAT |
| Actin F | AGAAAATCTGGCACCACACC |
| Actin R | AGAGGCGTACAGGGATAGCA |
| CERS1 F | AGGAGGAGACGATGAGGATGA |
| CERS1 R | ACGCTACGCTATACATGGACAC |
| DEGS2 F | CGGCGCAAGGAGATACTGG |
| DEGS2 R | GTTGTGCGAGATGTCGTGGA |
| DHCR7 F | GCTGCAAAATCGCAACCCAA |
| DHCR7 R | GCTCGCCAGTGAAAACCAGT |
| GLTP F | AAGCTGTGTACGACACCAACC |
| GLTP R | GCGTTGACACGGATGAGGT |
| GOLM1 F | TGGCCTGCATCATCGTCTTG |
| GOLM1 R | CCCTGGAACTCGTTCTTCTTCA |
| GRASP55 F | AACACAGACACTGATAACTGTCG |
| GRASP55 R | GCGTGTAGGTATTCGATGCAAA |
| HMGCS1 F | CATTAGACCGCTGCTATTCTGTC |
| HMGCS1 R | TTCAGCAACATCCGAGCTAGA |
| KDSR F | GTGGCATCGGGAAGTGCAT |
| KDSR R | AAGCACCACCTGTTTGTCATT |
| MT- CYB F | ATCACTCGAGACGTAAATTATGGCT |
| MT- CYB R | TGAACTAGGTCTGTCCCAATGTATG |
| MT- ND1 F | CCACCTCTAGCCTAGCCGTTTA |
| MT- ND1 R | GGGTCATGATGGCAGGAGTAAT |
| MT- ND5 F | TCTACCCTAGCATCACACACCG |
| MT- ND5 R | GTTGAGGTGATGATGGAGGTGG |
| ORMDL1 F | TGACCAGGGTAAAGCAAGGC |
| ORMDL1 R | CCGAACACCATGTAGTTGTGG |
| ORMDL3 F | GAGGCTGCTAACCCACTGG |
| ORMDL3 R | GGTGAGGAAGTACAGCACGAT |
| RPLPO F | TGGTCATCCAGGTGTTCGA |
| RPLPO R | ACAGACACTGGCAACATTGCGG |
| SCARB1 F | CCTATCCCCTTCTATCTCTCCG |
| SCARB1 R | GGATGTTGGGCATGACGATGT |
| SCD F | GCCCCTCTACTTGGAAGACGA |
| SCD R | AAGTGATCCCATACAGGGCTC |

| **Gene** | **Primer sequence** |
| --- | --- |
| SDHA F | CATGCTGCCGTGTTCCGTGTGGG |
| SDHA R | GGACAGGGTGTGCTTCCTCCAGTGCTCC |
| SOAT1 F | CAAGGCGCTCTCTCTTAGATG |
| SOAT1 R | GGTCCAAACAACGGTAGGAAA |
| SOAT2 F | ATGGAAACACTGAGACGCACA |
| SOAT2 R | GGTAGGATTGTATAGCCTCCCG |
| SPTLC1 F | TGGTTGCCACTCTTCAATCAG |
| SPTLC1 R | GGTGGAGATGGTACAGGCG |
| SPTLC2 F | AACGGGGAAGTACGGAAC |
| SPTLC2 R | CCCCACATACGTGAGCACAG |
| SPTLC3 F | GGAATTGGAACCCTGTTTGGC |
| SPTLC3 R | GTCTCTGATTCGCATGTAAAGGT |
| SPTSSB F | GCTGTGCTGTTTTAGAGCCCT |
| SPTSSB R | CCAGGCGAATGTGGATTGG |
| SREBP2 F | AACGGTCATTCACCCAGGTC |
| SREBP2 R | GGCTGAAGAATAGGAGTTGCC |
| VPS51 F | GCTATTTTGCGCTGGTGGAG |
| VPS51 R | AATGTGGCTCAGGATGGAGC |
| VPS53 F | TGTTCCCAACCGAGCAATCTC |
| VPS53 R | ACGTTCGTCTGACCTCTTACA |
| VPS54 F | GGAAAAGGAAATCGTGATGCAGC |
| VPS54 R | CTGGTGAGCAATGTTTACTTCCA |

**Supplementary Table 2. The effects of GOLM1 knockdown on total lipid classes and species concentrations**

| **Lipids** | **NT siRNA (Mean value with SD)** | | | **siGOLM1 (Mean value with SD)** | | | **P value** | **Q value** |
| --- | --- | --- | --- | --- | --- | --- | --- | --- |
| CE | 26.058 | **±** | 2.496 | 33.207 | **±** | 1.762 | 0.015873 | 0.023765 |
| CE 14:0 | 0.844 | **±** | 0.073 | 1.092 | **±** | 0.088 | 0.015873 | 0.023765 |
| CE 15:0 | 0.349 | **±** | 0.049 | 0.44 | **±** | 0.04 | 0.015873 | 0.023765 |
| CE 16:0 | 5.527 | **±** | 0.558 | 7.546 | **±** | 0.424 | 0.007937 | 0.016245 |
| CE 16:1 | 2.748 | **±** | 0.23 | 2.976 | **±** | 0.184 | 0.222222 | 0.18057 |
| CE 16:2 | 0.129 | **±** | 0.01 | 0.135 | **±** | 0.005 | 0.198413 | 0.171569 |
| CE 18:0 | 1.013 | **±** | 0.125 | 2.295 | **±** | 0.354 | 0.007937 | 0.016245 |
| CE 18:1 | 11.697 | **±** | 1.093 | 14.112 | **±** | 0.585 | 0.015873 | 0.023765 |
| CE 18:2 | 1.185 | **±** | 0.109 | 1.388 | **±** | 0.076 | 0.031746 | 0.041734 |
| CE 18:3 | 0.111 | **±** | 0.011 | 0.149 | **±** | 0.012 | 0.015873 | 0.023765 |
| CE 20:1 | 0.438 | **±** | 0.038 | 0.484 | **±** | 0.031 | 0.055556 | 0.061954 |
| CE 20:2 | 0.336 | **±** | 0.024 | 0.362 | **±** | 0.02 | 0.095238 | 0.093902 |
| CE 20:3 | 0.247 | **±** | 0.021 | 0.277 | **±** | 0.015 | 0.095238 | 0.093902 |
| CE 20:4 | 0.317 | **±** | 0.044 | 0.458 | **±** | 0.043 | 0.015873 | 0.023765 |
| CE 20:5 | 0.083 | **±** | 0.012 | 0.13 | **±** | 0.018 | 0.015873 | 0.023765 |
| CE 22:6 | 1.034 | **±** | 0.116 | 1.364 | **±** | 0.133 | 0.015873 | 0.023765 |
| Cer | 0.677 | **±** | 0.065 | 1.157 | **±** | 0.114 | 0.007937 | 0.016245 |
| Cer 18:1;O2/16:0 | 0.223 | **±** | 0.022 | 0.439 | **±** | 0.038 | 0.007937 | 0.016245 |
| Cer 18:1;O2/18:0 | 0.029 | **±** | 0.002 | 0.057 | **±** | 0.005 | 0.007937 | 0.016245 |
| Cer 18:1;O2/22:0 | 0.065 | **±** | 0.005 | 0.117 | **±** | 0.017 | 0.007937 | 0.016245 |
| Cer 18:1;O2/22:1 | 0.005 | **±** | 0.001 | 0.008 | **±** | 0.001 | 0.007937 | 0.016245 |
| Cer 18:1;O2/23:0 | 0.027 | **±** | 0.005 | 0.041 | **±** | 0.007 | 0.02381 | 0.035 |
| Cer 18:1;O2/24:0 | 0.186 | **±** | 0.021 | 0.293 | **±** | 0.038 | 0.007937 | 0.016245 |
| Cer 18:1;O2/24:1 | 0.142 | **±** | 0.013 | 0.201 | **±** | 0.012 | 0.007937 | 0.016245 |
| **CerP 18:1;O2** | **10.062** | **±** | **3.324** | **33.81** | **±** | **4.729** | **0.007937** | **0.016245** |
| **CerP 18:1;O2/16:0** | **3.766** | **±** | **0.403** | **7.772** | **±** | **0.977** | **0.007937** | **0.016245** |
| **CerP 18:1;O2/22:0** | **0** | **±** | **0** | **3.466** | **±** | **2.209** | **0.047619** | **0.060156** |
| **CerP 18:1;O2/24:0** | **1.472** | **±** | **2.107** | **6.778** | **±** | **1.701** | **0.015873** | **0.023765** |
| **CerP 18:1;O2/24:1** | **4.826** | **±** | **1.306** | **15.792** | **±** | **1.927** | **0.007937** | **0.016245** |
| DG | 7.877 | **±** | 1.103 | 7.09 | **±** | 0.627 | 0.309524 | 0.239474 |
| DG 32:0 | 0.393 | **±** | 0.062 | 0.432 | **±** | 0.033 | 0.309524 | 0.239474 |
| DG 32:1 | 0.815 | **±** | 0.107 | 0.659 | **±** | 0.078 | 0.055556 | 0.061954 |
| DG 32:2 | 0.181 | **±** | 0.022 | 0.08 | **±** | 0.027 | 0.007937 | 0.016245 |
| DG 34:1 | 2.083 | **±** | 0.305 | 2.075 | **±** | 0.183 | 0.690476 | 0.452024 |
| DG 34:2 | 1.079 | **±** | 0.126 | 0.608 | **±** | 0.119 | 0.007937 | 0.016245 |
| DG 36:1 | 0.605 | **±** | 0.119 | 1.066 | **±** | 0.147 | 0.007937 | 0.016245 |
| DG 36:2 | 1.719 | **±** | 0.247 | 1.398 | **±** | 0.144 | 0.031746 | 0.041734 |
| DG 36:3 | 0.34 | **±** | 0.037 | 0.213 | **±** | 0.052 | 0.015873 | 0.023765 |
| DG 36:4 | 0.083 | **±** | 0.025 | 0.05 | **±** | 0.011 | 0.055556 | 0.061954 |
| DG 38:2 | 0.121 | **±** | 0.022 | 0.132 | **±** | 0.009 | 0.150794 | 0.133242 |
| DG 38:3 | 0.286 | **±** | 0.036 | 0.24 | **±** | 0.042 | 0.222222 | 0.18057 |
| DG 38:4 | 0.171 | **±** | 0.022 | 0.136 | **±** | 0.024 | 0.039683 | 0.051747 |
| FC | 86.253 | **±** | 10.872 | 96.077 | **±** | 10.429 | 0.309524 | 0.239474 |
| **Hex2Cer 18:1;O2** | **176.42** | **±** | **25.903** | **394.046** | **±** | **36.419** | **0.007937** | **0.016245** |
| **Hex2Cer 18:1;O2/16:0** | **97.298** | **±** | **13.396** | **230.268** | **±** | **18.439** | **0.007937** | **0.016245** |
| **Hex2Cer 18:1;O2/22:0** | **23.016** | **±** | **3.73** | **50.872** | **±** | **7.753** | **0.007937** | **0.016245** |
| **Hex2Cer 18:1;O2/23:0** | **6.352** | **±** | **0.816** | **11.736** | **±** | **1.275** | **0.007937** | **0.016245** |
| **Hex2Cer 18:1;O2/24:0** | **28.926** | **±** | **5.355** | **64.262** | **±** | **8.163** | **0.007937** | **0.016245** |
| **Hex2Cer 18:1;O2/24:1** | **20.826** | **±** | **2.845** | **36.914** | **±** | **3.121** | **0.007937** | **0.016245** |
| HexCer 18:1;O2 | 0.326 | **±** | 0.035 | 0.972 | **±** | 0.114 | 0.007937 | 0.016245 |
| HexCer 18:1;O2/16:0 | 0.108 | **±** | 0.011 | 0.412 | **±** | 0.037 | 0.007937 | 0.016245 |
| HexCer 18:1;O2/18:0 | 0.011 | **±** | 0.001 | 0.042 | **±** | 0.007 | 0.007937 | 0.016245 |
| HexCer 18:1;O2/22:0 | 0.047 | **±** | 0.007 | 0.157 | **±** | 0.03 | 0.007937 | 0.016245 |
| HexCer 18:1;O2/23:0 | 0.019 | **±** | 0.003 | 0.035 | **±** | 0.003 | 0.007937 | 0.016245 |
| HexCer 18:1;O2/24:0 | 0.099 | **±** | 0.011 | 0.237 | **±** | 0.035 | 0.007937 | 0.016245 |
| HexCer 18:1;O2/24:1 | 0.043 | **±** | 0.007 | 0.089 | **±** | 0.008 | 0.007937 | 0.016245 |
| LPC | 0.85 | **±** | 0.15 | 0.667 | **±** | 0.106 | 0.055556 | 0.061954 |
| LPC 16:0 | 0.207 | **±** | 0.031 | 0.181 | **±** | 0.029 | 0.34127 | 0.261532 |
| LPC 16:1 | 0.077 | **±** | 0.014 | 0.038 | **±** | 0.002 | 0.007937 | 0.016245 |
| LPC 18:0 | 0.201 | **±** | 0.03 | 0.207 | **±** | 0.05 | >0.999999 | 0.617176 |
| LPC 18:1 | 0.302 | **±** | 0.064 | 0.195 | **±** | 0.022 | 0.007937 | 0.016245 |
| LPC 18:2 | 0.017 | **±** | 0.004 | 0.008 | **±** | 0.001 | 0.007937 | 0.016245 |
| LPC 20:3 | 0.023 | **±** | 0.004 | 0.013 | **±** | 0.002 | 0.007937 | 0.016245 |
| LPC 20:4 | 0.007 | **±** | 0.002 | 0.006 | **±** | 0.001 | 0.531746 | 0.378419 |
| LPC 20:5 | 0.003 | **±** | 0.001 | 0.002 | **±** | 0.001 | >0.999999 | 0.617176 |
| LPC 22:4 | 0.002 | **±** | 0.001 | 0.004 | **±** | 4.47E-04 | 0.02381 | 0.035 |
| LPC 22:5 | 0.004 | **±** | 4.47E-04 | 0.005 | **±** | 0.001 | 0.119048 | 0.114583 |
| LPC 22:6 | 0.006 | **±** | 0.001 | 0.008 | **±** | 0.002 | 0.079365 | 0.08613 |
| LPE | 0.588 | **±** | 0.098 | 0.347 | **±** | 0.042 | 0.007937 | 0.016245 |
| LPE 16:0 | 0.094 | **±** | 0.016 | 0.061 | **±** | 0.006 | 0.007937 | 0.016245 |
| LPE 18:0 | 0.246 | **±** | 0.037 | 0.176 | **±** | 0.02 | 0.007937 | 0.016245 |
| LPE 18:1 | 0.181 | **±** | 0.033 | 0.089 | **±** | 0.009 | 0.007937 | 0.016245 |
| LPE 20:1 | 0.014 | **±** | 0.008 | 0 | **±** | 0 | 0.047619 | 0.060156 |
| LPE 20:3 | 0.029 | **±** | 0.003 | 0.004 | **±** | 0.008 | 0.007937 | 0.016245 |
| LPE 20:4 | 0.021 | **±** | 0.002 | 0.006 | **±** | 0.009 | 0.007937 | 0.016245 |
| LPE 22:6 | 0.003 | **±** | 0.008 | 0.011 | **±** | 0.01 | 0.285714 | 0.227586 |
| PC | 76.795 | **±** | 9.864 | 86.687 | **±** | 8.836 | 0.095238 | 0.093902 |
| PC 30:0 | 1.195 | **±** | 0.209 | 1.095 | **±** | 0.111 | 0.690476 | 0.452024 |
| PC 30:1 | 0.577 | **±** | 0.071 | 0.691 | **±** | 0.068 | 0.055556 | 0.061954 |
| PC 32:0 | 3.572 | **±** | 0.591 | 3.247 | **±** | 0.235 | 0.84127 | 0.537681 |
| PC 32:1 | 11.45 | **±** | 1.382 | 10.743 | **±** | 0.615 | 0.690476 | 0.452024 |
| PC 32:2 | 1.62 | **±** | 0.125 | 1.476 | **±** | 0.083 | 0.095238 | 0.093902 |
| PC 34:0 | 1.027 | **±** | 0.201 | 1.531 | **±** | 0.304 | 0.031746 | 0.041734 |
| PC 34:1 | 20.315 | **±** | 2.91 | 25.917 | **±** | 2.984 | 0.031746 | 0.041734 |
| PC 34:2 | 7.258 | **±** | 0.809 | 7.128 | **±** | 0.504 | 0.84127 | 0.537681 |
| PC 34:3 | 0.775 | **±** | 0.067 | 0.719 | **±** | 0.036 | 0.095238 | 0.093902 |
| PC 36:0 | 0.141 | **±** | 0.022 | 0.234 | **±** | 0.06 | 0.031746 | 0.041734 |
| PC 36:1 | 6.43 | **±** | 0.965 | 8.769 | **±** | 1.75 | 0.055556 | 0.061954 |
| PC 36:2 | 10.816 | **±** | 1.479 | 11.719 | **±** | 1.379 | 0.547619 | 0.378419 |
| PC 36:3 | 2.969 | **±** | 0.253 | 3.014 | **±** | 0.164 | 0.690476 | 0.452024 |
| PC 36:4 | 0.791 | **±** | 0.058 | 0.958 | **±** | 0.054 | 0.015873 | 0.023765 |
| PC 36:5 | 0.223 | **±** | 0.024 | 0.352 | **±** | 0.047 | 0.007937 | 0.016245 |
| PC 38:1 | 0.294 | **±** | 0.051 | 0.397 | **±** | 0.095 | 0.055556 | 0.061954 |
| PC 38:2 | 1.194 | **±** | 0.186 | 1.271 | **±** | 0.178 | 0.690476 | 0.452024 |
| PC 38:3 | 2.149 | **±** | 0.192 | 2.382 | **±** | 0.149 | 0.095238 | 0.093902 |
| PC 38:4 | 2.096 | **±** | 0.141 | 2.133 | **±** | 0.114 | 0.84127 | 0.537681 |
| PC 38:5 | 0.778 | **±** | 0.054 | 0.955 | **±** | 0.114 | 0.015873 | 0.023765 |
| PC 38:6 | 0.562 | **±** | 0.053 | 1.045 | **±** | 0.224 | 0.007937 | 0.016245 |
| PC 40:5 | 0.193 | **±** | 0.019 | 0.232 | **±** | 0.032 | 0.095238 | 0.093902 |
| PC 40:6 | 0.372 | **±** | 0.037 | 0.678 | **±** | 0.173 | 0.007937 | 0.016245 |
| PC O | 12.067 | **±** | 1.785 | 13.974 | **±** | 1.949 | 0.095238 | 0.093902 |
| PC O-32:0 | 0.98 | **±** | 0.169 | 0.939 | **±** | 0.133 | >0.999999 | 0.617176 |
| PC O-32:1 | 0.777 | **±** | 0.106 | 0.846 | **±** | 0.083 | 0.420635 | 0.306381 |
| PC O-34:0 | 1.064 | **±** | 0.18 | 1.144 | **±** | 0.18 | 0.690476 | 0.452024 |
| PC O-34:1 | 3.333 | **±** | 0.482 | 3.813 | **±** | 0.5 | 0.150794 | 0.133242 |
| PC O-34:2 | 1.01 | **±** | 0.125 | 1.078 | **±** | 0.094 | 0.420635 | 0.306381 |
| PC O-36:1 | 2.269 | **±** | 0.366 | 3.047 | **±** | 0.584 | 0.055556 | 0.061954 |
| PC O-36:2 | 1.984 | **±** | 0.295 | 2.28 | **±** | 0.328 | 0.150794 | 0.133242 |
| PC O-36:4 | 0.101 | **±** | 0.011 | 0.144 | **±** | 0.011 | 0.007937 | 0.016245 |
| PC O-36:5 | 0.061 | **±** | 0.009 | 0.094 | **±** | 0.014 | 0.007937 | 0.016245 |
| PC O-38:4 | 0.346 | **±** | 0.029 | 0.387 | **±** | 0.017 | 0.095238 | 0.093902 |
| PC O-38:5 | 0.142 | **±** | 0.017 | 0.202 | **±** | 0.027 | 0.007937 | 0.016245 |
| PE | 28.638 | **±** | 3.503 | 23.881 | **±** | 1.606 | 0.015873 | 0.023765 |
| PE 32:1 | 0.268 | **±** | 0.035 | 0.176 | **±** | 0.011 | 0.007937 | 0.016245 |
| PE 34:1 | 1.195 | **±** | 0.18 | 0.958 | **±** | 0.067 | 0.015873 | 0.023765 |
| PE 34:2 | 0.953 | **±** | 0.128 | 0.498 | **±** | 0.02 | 0.007937 | 0.016245 |
| PE 34:3 | 0.11 | **±** | 0.012 | 0.061 | **±** | 0.004 | 0.007937 | 0.016245 |
| PE 36:1 | 0.903 | **±** | 0.152 | 0.903 | **±** | 0.16 | >0.999999 | 0.617176 |
| PE 36:2 | 2.239 | **±** | 0.361 | 1.451 | **±** | 0.104 | 0.007937 | 0.016245 |
| PE 36:3 | 1.095 | **±** | 0.122 | 0.592 | **±** | 0.044 | 0.007937 | 0.016245 |
| PE 36:4 | 0.577 | **±** | 0.061 | 0.466 | **±** | 0.022 | 0.007937 | 0.016245 |
| PE 36:5 | 0.151 | **±** | 0.02 | 0.122 | **±** | 0.006 | 0.007937 | 0.016245 |
| PE 38:2 | 0.419 | **±** | 0.058 | 0.313 | **±** | 0.03 | 0.007937 | 0.016245 |
| PE 38:3 | 4.974 | **±** | 0.548 | 3.302 | **±** | 0.232 | 0.007937 | 0.016245 |
| PE 38:4 | 8.18 | **±** | 0.875 | 7.511 | **±** | 0.568 | 0.420635 | 0.306381 |
| PE 38:5 | 3.868 | **±** | 0.468 | 3.52 | **±** | 0.212 | 0.095238 | 0.093902 |
| PE 38:6 | 1.323 | **±** | 0.173 | 1.477 | **±** | 0.108 | 0.222222 | 0.18057 |
| PE 40:3 | 0.124 | **±** | 0.019 | 0.109 | **±** | 0.01 | 0.238095 | 0.1925 |
| PE 40:4 | 0.259 | **±** | 0.03 | 0.19 | **±** | 0.011 | 0.007937 | 0.016245 |
| PE 40:5 | 0.416 | **±** | 0.047 | 0.36 | **±** | 0.048 | 0.150794 | 0.133242 |
| PE 40:6 | 1.583 | **±** | 0.231 | 1.872 | **±** | 0.324 | 0.150794 | 0.133242 |
| PE P | 0.592 | **±** | 0.084 | 0.679 | **±** | 0.069 | 0.150794 | 0.133242 |
| PE P-16:0/16:1 | 0.017 | **±** | 0.004 | 0.014 | **±** | 0.002 | 0.214286 | 0.18057 |
| PE P-16:0/18:1 | 0.064 | **±** | 0.009 | 0.047 | **±** | 0.008 | 0.047619 | 0.060156 |
| PE P-16:0/18:2 | 0.012 | **±** | 0.003 | 0.009 | **±** | 0.001 | 0.087302 | 0.093902 |
| PE P-16:0/20:3 | 0.089 | **±** | 0.01 | 0.058 | **±** | 0.007 | 0.007937 | 0.016245 |
| PE P-16:0/20:4 | 0.061 | **±** | 0.009 | 0.071 | **±** | 0.007 | 0.111111 | 0.107585 |
| PE P-16:0/20:5 | 0.024 | **±** | 0.004 | 0.029 | **±** | 0.006 | 0.222222 | 0.18057 |
| PE P-16:0/22:5 | 0.009 | **±** | 0.003 | 0.012 | **±** | 0.001 | 0.174603 | 0.151792 |
| PE P-16:0/22:6 | 0.047 | **±** | 0.009 | 0.129 | **±** | 0.019 | 0.007937 | 0.016245 |
| PE P-18:0/18:1 | 0.021 | **±** | 0.006 | 0.019 | **±** | 0.003 | 0.650794 | 0.445904 |
| PE P-18:0/20:3 | 0.044 | **±** | 0.007 | 0.027 | **±** | 0.004 | 0.007937 | 0.016245 |
| PE P-18:0/20:4 | 0.021 | **±** | 0.001 | 0.033 | **±** | 0.006 | 0.007937 | 0.016245 |
| PE P-18:0/22:5 | 0.005 | **±** | 0.002 | 0.008 | **±** | 0.002 | 0.055556 | 0.061954 |
| PE P-18:0/22:6 | 0.022 | **±** | 0.005 | 0.052 | **±** | 0.009 | 0.007937 | 0.016245 |
| PE P-18:1/16:1 | 0.015 | **±** | 0.004 | 0.011 | **±** | 0.003 | 0.095238 | 0.093902 |
| PE P-18:1/18:1 | 0.04 | **±** | 0.007 | 0.031 | **±** | 0.003 | 0.007937 | 0.016245 |
| PE P-18:1/20:3 | 0.041 | **±** | 0.007 | 0.028 | **±** | 0.002 | 0.007937 | 0.016245 |
| PE P-18:1/20:4 | 0.029 | **±** | 0.005 | 0.035 | **±** | 0.005 | 0.071429 | 0.078041 |
| PE P-18:1/20:5 | 0.011 | **±** | 0.003 | 0.014 | **±** | 0.002 | 0.103175 | 0.100502 |
| PE P-18:1/22:6 | 0.02 | **±** | 0.003 | 0.052 | **±** | 0.008 | 0.007937 | 0.016245 |
| PG | 0.499 | **±** | 0.066 | 0.57 | **±** | 0.053 | 0.142857 | 0.133242 |
| PG 32:1 | 0.016 | **±** | 0.004 | 0.016 | **±** | 0.003 | 0.84127 | 0.537681 |
| PG 34:1 | 0.294 | **±** | 0.038 | 0.395 | **±** | 0.037 | 0.015873 | 0.023765 |
| PG 34:2 | 0.036 | **±** | 0.003 | 0.031 | **±** | 0.003 | 0.063492 | 0.07032 |
| PG 36:1 | 0.115 | **±** | 0.019 | 0.091 | **±** | 0.014 | 0.015873 | 0.023765 |
| PG 36:2 | 0.039 | **±** | 0.005 | 0.036 | **±** | 0.002 | 0.460317 | 0.333782 |
| PI | 41.712 | **±** | 4.778 | 41.471 | **±** | 2.581 | >0.999999 | 0.617176 |
| PI 34:1 | 0.58 | **±** | 0.131 | 0.699 | **±** | 0.108 | 0.150794 | 0.133242 |
| PI 34:2 | 0.317 | **±** | 0.068 | 0.234 | **±** | 0.022 | 0.031746 | 0.041734 |
| PI 36:1 | 0.329 | **±** | 0.116 | 0.7 | **±** | 0.164 | 0.015873 | 0.023765 |
| PI 36:2 | 1.996 | **±** | 0.426 | 1.75 | **±** | 0.137 | 0.690476 | 0.452024 |
| PI 36:3 | 3.239 | **±** | 0.274 | 2.72 | **±** | 0.249 | 0.031746 | 0.041734 |
| PI 36:4 | 0.754 | **±** | 0.104 | 0.72 | **±** | 0.1 | 0.690476 | 0.452024 |
| PI 38:2 | 0.846 | **±** | 0.18 | 1.138 | **±** | 0.158 | 0.031746 | 0.041734 |
| PI 38:3 | 18.424 | **±** | 1.488 | 17.93 | **±** | 1.627 | 0.420635 | 0.306381 |
| PI 38:4 | 10.315 | **±** | 1.256 | 9.96 | **±** | 1.093 | 0.690476 | 0.452024 |
| PI 38:5 | 1.796 | **±** | 0.251 | 1.485 | **±** | 0.268 | 0.309524 | 0.239474 |
| PI 38:6 | 0.407 | **±** | 0.08 | 0.502 | **±** | 0.102 | 0.150794 | 0.133242 |
| PI 40:3 | 0.56 | **±** | 0.065 | 0.664 | **±** | 0.045 | 0.055556 | 0.061954 |
| PI 40:4 | 0.6 | **±** | 0.078 | 0.526 | **±** | 0.055 | 0.222222 | 0.18057 |
| PI 40:5 | 0.426 | **±** | 0.088 | 0.534 | **±** | 0.115 | 0.095238 | 0.093902 |
| PI 40:6 | 1.122 | **±** | 0.217 | 1.909 | **±** | 0.577 | 0.055556 | 0.061954 |
| PS | 20.698 | **±** | 2.987 | 20.582 | **±** | 1.784 | 0.84127 | 0.537681 |
| PS 32:1 | 0.42 | **±** | 0.056 | 0.275 | **±** | 0.017 | 0.007937 | 0.016245 |
| PS 34:1 | 2.03 | **±** | 0.284 | 1.748 | **±** | 0.099 | 0.031746 | 0.041734 |
| PS 34:2 | 0.406 | **±** | 0.063 | 0.287 | **±** | 0.022 | 0.007937 | 0.016245 |
| PS 36:1 | 3.128 | **±** | 0.502 | 3.178 | **±** | 0.346 | 0.690476 | 0.452024 |
| PS 36:2 | 1.424 | **±** | 0.231 | 1.222 | **±** | 0.111 | 0.150794 | 0.133242 |
| PS 36:3 | 0.514 | **±** | 0.05 | 0.388 | **±** | 0.028 | 0.007937 | 0.016245 |
| PS 36:4 | 0.145 | **±** | 0.019 | 0.143 | **±** | 0.011 | >0.999999 | 0.617176 |
| PS 38:1 | 0.274 | **±** | 0.04 | 0.289 | **±** | 0.025 | 0.420635 | 0.306381 |
| PS 38:2 | 0.585 | **±** | 0.1 | 0.453 | **±** | 0.053 | 0.015873 | 0.023765 |
| PS 38:3 | 3.484 | **±** | 0.384 | 3.183 | **±** | 0.217 | 0.095238 | 0.093902 |
| PS 38:4 | 2.191 | **±** | 0.231 | 3.351 | **±** | 0.414 | 0.007937 | 0.016245 |
| PS 38:5 | 0.582 | **±** | 0.052 | 0.822 | **±** | 0.095 | 0.007937 | 0.016245 |
| PS 38:6 | 0.361 | **±** | 0.065 | 0.286 | **±** | 0.021 | 0.047619 | 0.060156 |
| PS 40:3 | 0.308 | **±** | 0.051 | 0.278 | **±** | 0.023 | 0.309524 | 0.239474 |
| PS 40:4 | 0.232 | **±** | 0.038 | 0.245 | **±** | 0.021 | 0.34127 | 0.261532 |
| PS 40:5 | 0.543 | **±** | 0.087 | 0.611 | **±** | 0.084 | 0.222222 | 0.18057 |
| PS 40:6 | 3.495 | **±** | 0.65 | 3.356 | **±** | 0.59 | >0.999999 | 0.617176 |
| PS 40:7 | 0.462 | **±** | 0.102 | 0.323 | **±** | 0.036 | 0.007937 | 0.016245 |
| PS 42:6 | 0.116 | **±** | 0.029 | 0.142 | **±** | 0.024 | 0.174603 | 0.151792 |
| SM | 11.102 | **±** | 1.684 | 12.098 | **±** | 1.37 | 0.547619 | 0.378419 |
| SM 32:1;O2 | 0.275 | **±** | 0.036 | 0.292 | **±** | 0.027 | 0.420635 | 0.306381 |
| SM 33:1;O2 | 0.349 | **±** | 0.052 | 0.368 | **±** | 0.035 | 0.547619 | 0.378419 |
| SM 34:0;O2 | 1.072 | **±** | 0.179 | 1.262 | **±** | 0.228 | 0.222222 | 0.18057 |
| SM 34:1;O2 | 4.809 | **±** | 0.717 | 5.12 | **±** | 0.483 | 0.420635 | 0.306381 |
| SM 34:2;O2 | 0.336 | **±** | 0.046 | 0.363 | **±** | 0.03 | 0.246032 | 0.197927 |
| SM 35:0;O2 | 0.048 | **±** | 0.006 | 0.066 | **±** | 0.014 | 0.055556 | 0.061954 |
| SM 35:1;O2 | 0.157 | **±** | 0.023 | 0.177 | **±** | 0.02 | 0.285714 | 0.227586 |
| SM 36:1;O2 | 0.423 | **±** | 0.071 | 0.476 | **±** | 0.065 | 0.309524 | 0.239474 |
| SM 36:2;O2 | 0.156 | **±** | 0.018 | 0.16 | **±** | 0.013 | 0.746032 | 0.486425 |
| SM 38:1;O2 | 0.168 | **±** | 0.029 | 0.179 | **±** | 0.032 | 0.634921 | 0.436879 |
| SM 38:2;O2 | 0.039 | **±** | 0.007 | 0.044 | **±** | 0.006 | 0.412698 | 0.306381 |
| SM 40:0;O2 | 0.174 | **±** | 0.044 | 0.274 | **±** | 0.063 | 0.031746 | 0.041734 |
| SM 40:1;O2 | 0.779 | **±** | 0.127 | 0.905 | **±** | 0.101 | 0.134921 | 0.129093 |
| SM 40:2;O2 | 0.204 | **±** | 0.034 | 0.22 | **±** | 0.025 | 0.420635 | 0.306381 |
| SM 42:1;O2 | 1.004 | **±** | 0.161 | 1.121 | **±** | 0.156 | 0.547619 | 0.378419 |
| SM 42:2;O2 | 0.929 | **±** | 0.135 | 0.9 | **±** | 0.087 | >0.999999 | 0.617176 |
| SM 42:3;O2 | 0.178 | **±** | 0.019 | 0.17 | **±** | 0.015 | 0.547619 | 0.378419 |
| **SPB 18:0;O2** | **8.948** | **±** | **0.775** | **14.328** | **±** | **3.592** | **0.015873** | **0.023765** |
| **SPB 18:0;O3** | **5.477** | **±** | **0.225** | **6.034** | **±** | **0.254** | **0.071429** | **0.078041** |
| **SPB 18:1;O2** | **27.068** | **±** | **2.344** | **40.112** | **±** | **9.317** | **0.015873** | **0.023765** |
| TG | 66.411 | **±** | 7.73 | 62.87 | **±** | 6.691 | 0.547619 | 0.378419 |
| TG 48:0 | 0.371 | **±** | 0.076 | 0.421 | **±** | 0.035 | 0.166667 | 0.146467 |
| TG 48:1 | 1.361 | **±** | 0.183 | 1.238 | **±** | 0.173 | 0.222222 | 0.18057 |
| TG 48:2 | 0.904 | **±** | 0.077 | 0.631 | **±** | 0.125 | 0.015873 | 0.023765 |
| TG 48:3 | 0.213 | **±** | 0.017 | 0.129 | **±** | 0.022 | 0.007937 | 0.016245 |
| TG 48:4 | 0.378 | **±** | 0.051 | 0.448 | **±** | 0.068 | 0.150794 | 0.133242 |
| TG 49:1 | 0.293 | **±** | 0.052 | 0.292 | **±** | 0.034 | >0.999999 | 0.617176 |
| TG 49:2 | 0.252 | **±** | 0.036 | 0.203 | **±** | 0.028 | 0.055556 | 0.061954 |
| TG 49:3 | 0.068 | **±** | 0.007 | 0.047 | **±** | 0.01 | 0.015873 | 0.023765 |
| TG 50:0 | 0.539 | **±** | 0.107 | 0.644 | **±** | 0.067 | 0.150794 | 0.133242 |
| TG 50:1 | 3.987 | **±** | 0.561 | 4.117 | **±** | 0.494 | 0.547619 | 0.378419 |
| TG 50:2 | 4.974 | **±** | 0.539 | 3.608 | **±** | 0.592 | 0.015873 | 0.023765 |
| TG 50:3 | 1.512 | **±** | 0.1 | 0.835 | **±** | 0.172 | 0.007937 | 0.016245 |
| TG 50:4 | 0.258 | **±** | 0.017 | 0.138 | **±** | 0.024 | 0.007937 | 0.016245 |
| TG 51:1 | 0.507 | **±** | 0.092 | 0.63 | **±** | 0.089 | 0.055556 | 0.061954 |
| TG 51:2 | 0.852 | **±** | 0.114 | 0.788 | **±** | 0.074 | 0.547619 | 0.378419 |
| TG 51:3 | 0.347 | **±** | 0.036 | 0.252 | **±** | 0.027 | 0.007937 | 0.016245 |
| TG 52:1 | 3.056 | **±** | 0.631 | 5.05 | **±** | 0.754 | 0.015873 | 0.023765 |
| TG 52:2 | 10.66 | **±** | 1.395 | 9.855 | **±** | 1.203 | 0.222222 | 0.18057 |
| TG 52:3 | 5.562 | **±** | 0.496 | 3.449 | **±** | 0.63 | 0.007937 | 0.016245 |
| TG 52:4 | 0.606 | **±** | 0.045 | 0.208 | **±** | 0.119 | 0.007937 | 0.016245 |
| TG 52:5 | 0.171 | **±** | 0.009 | 0.1 | **±** | 0.022 | 0.007937 | 0.016245 |
| TG 53:2 | 0.913 | **±** | 0.13 | 1.068 | **±** | 0.12 | 0.222222 | 0.18057 |
| TG 53:3 | 0.84 | **±** | 0.107 | 0.719 | **±** | 0.078 | 0.055556 | 0.061954 |
| TG 53:4 | 0.24 | **±** | 0.019 | 0.195 | **±** | 0.018 | 0.015873 | 0.023765 |
| TG 54:2 | 5.296 | **±** | 0.807 | 7.317 | **±** | 0.782 | 0.015873 | 0.023765 |
| TG 54:3 | 9.13 | **±** | 1.013 | 7.609 | **±** | 0.869 | 0.055556 | 0.061954 |
| TG 54:4 | 2.528 | **±** | 0.177 | 1.669 | **±** | 0.283 | 0.007937 | 0.016245 |
| TG 54:5 | 0.552 | **±** | 0.036 | 0.314 | **±** | 0.062 | 0.007937 | 0.016245 |
| TG 54:6 | 0.189 | **±** | 0.019 | 0.202 | **±** | 0.031 | 0.690476 | 0.452024 |
| TG 55:2 | 0.262 | **±** | 0.041 | 0.379 | **±** | 0.053 | 0.015873 | 0.023765 |
| TG 55:3 | 0.499 | **±** | 0.043 | 0.516 | **±** | 0.057 | 0.547619 | 0.378419 |
| TG 55:4 | 0.311 | **±** | 0.028 | 0.28 | **±** | 0.026 | 0.103175 | 0.100502 |
| TG 56:2 | 0.875 | **±** | 0.16 | 1.362 | **±** | 0.183 | 0.015873 | 0.023765 |
| TG 56:3 | 1.889 | **±** | 0.218 | 2.026 | **±** | 0.18 | 0.222222 | 0.18057 |
| TG 56:4 | 1.857 | **±** | 0.152 | 1.626 | **±** | 0.177 | 0.095238 | 0.093902 |
| TG 56:5 | 1.066 | **±** | 0.065 | 0.786 | **±** | 0.127 | 0.015873 | 0.023765 |
| TG 56:6 | 0.324 | **±** | 0.026 | 0.346 | **±** | 0.037 | 0.388889 | 0.295227 |
| TG 56:7 | 0.498 | **±** | 0.044 | 0.599 | **±** | 0.061 | 0.031746 | 0.041734 |
| TG 58:2 | 0.288 | **±** | 0.048 | 0.391 | **±** | 0.065 | 0.031746 | 0.041734 |
| TG 58:3 | 0.35 | **±** | 0.043 | 0.39 | **±** | 0.033 | 0.150794 | 0.133242 |
| TG 58:4 | 0.089 | **±** | 0.038 | 0.135 | **±** | 0.078 | 0.5 | 0.360938 |
| TG 58:5 | 0.424 | **±** | 0.018 | 0.348 | **±** | 0.046 | 0.055556 | 0.061954 |
| TG 58:6 | 0.264 | **±** | 0.024 | 0.279 | **±** | 0.028 | 0.388889 | 0.295227 |
| TG 58:7 | 0.407 | **±** | 0.03 | 0.614 | **±** | 0.073 | 0.007937 | 0.016245 |
| TG 60:6 | 0.1 | **±** | 0.007 | 0.112 | **±** | 0.012 | 0.142857 | 0.133242 |
| TG 60:7 | 0.135 | **±** | 0.014 | 0.236 | **±** | 0.044 | 0.007937 | 0.016245 |
| TG 60:8 | 0.213 | **±** | 0.021 | 0.27 | **±** | 0.033 | 0.031746 | 0.041734 |

The data represents mean ± SD, significance is calculated by Mann Whitney (p-value) with the false discovery rate of 5% (q-value). The highlighted (**bold**) lipid classes and species are expressed in pmol/mg protein and the rest in nmol/mg protein scale. CE, cholesterol ester; Cer, ceramide; CerP, ceramide phosphate; DG, diacylglycerol; Hex2Cer, dihexosylceramide; HexCer, hexosylceramide; LPC, lysophosphatidylcholine; LPE, lysophosphatidylethanolamine; PC, phosphatidylcholine; PE, phosphatidylethanolamine; PE-P, phosphatidylethanolamine plasmalogen; PG, phosphatidylglycerol; PI, phosphatidylinositol; PS, phosphatidylserine; SM, sphingomyelin; SPB, sphingosine or sphinganine; TG, triacylglycerol.

**Supplementary Figure 1**. Sphingolipid synthesis by [^3^H]serine labelling in GOLM1 knockdown cells, Dashed (---) line represents controls. Radiolabelling measurements of hexosyl ceramides (Hex Cer), ceramides (Cer) and sphinganine (SN) are combined here. SM, sphingomyelin.


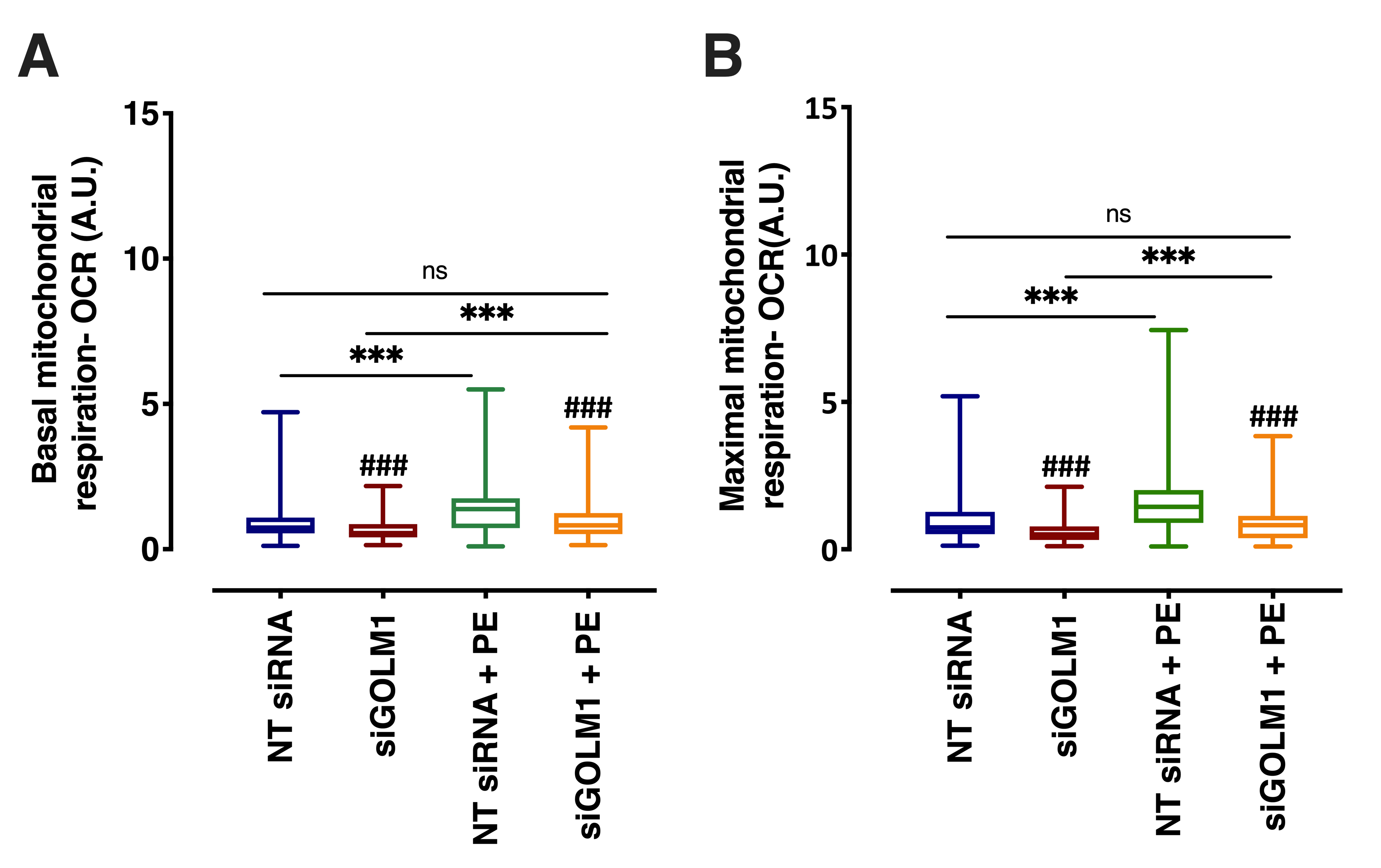


**Supplementary Figure 2.** PE loading alters mitochondrial respiration (OCR) in GOLM1 knockdown and control Huh-7 cells. (A) Basal and (B) Maximal mitochondrial respiration (OCR) with and without PE loading. Data is represented as mean ± SD, from four experiments each with multiple replicates. ***, ^###^ P_adj_ < 0.001. ***** represents significance between PE treated and untreated cells transfected with the same siRNA. **#** represents significance between NT- and siGOLM1-transfected cells in PE treated or untreated conditions.

**Full western blots:**

1. **Full blot of Figure 2B- Endogenous GOLM1 expression in HepG2 and Huh-7 cells.**


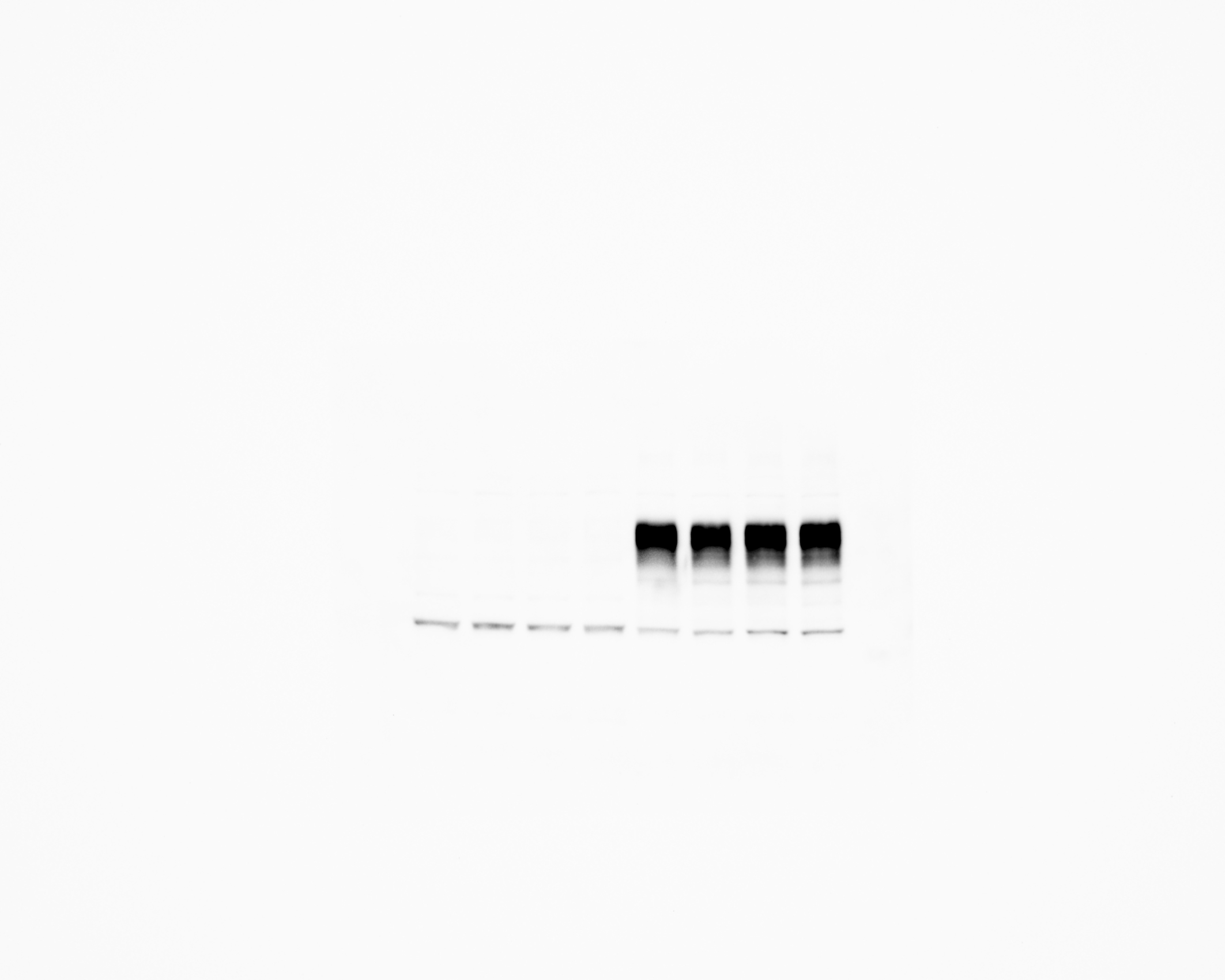


HepG2

Huh-7

**GOLM1**

**100kDa**

**75kDa**

1. **Full blot of Figure 2F- GOLM1 knockdown at protein level in Huh-7 cells.**


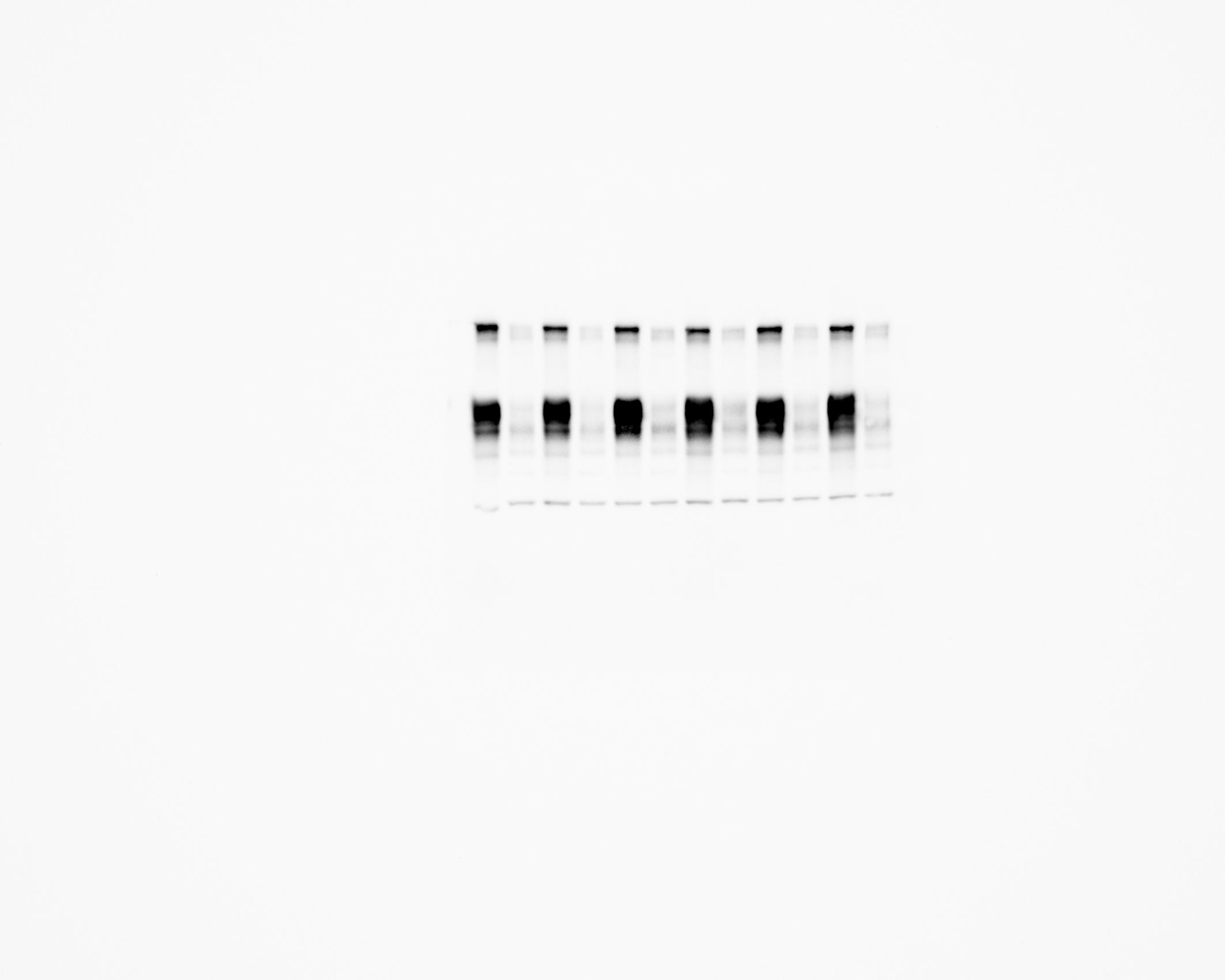


**GOLM1**

**100kDa**

**75kDa**

**siGOLM1**

**NT siRNA**

**NT siRNA**

**siGOLM1**

**siGOLM1**

**NT siRNA**

1. **Full blot of Figure 9C- ORMDL3 expression in GOLM1 knockdown Huh-7 cells.**

**siGOLM1**

**NT siRNA**

**NT siRNA**

**siGOLM1**

**siGOLM1**

**NT siRNA**

**20kDa**

**15kDa**

**ORMDL3**


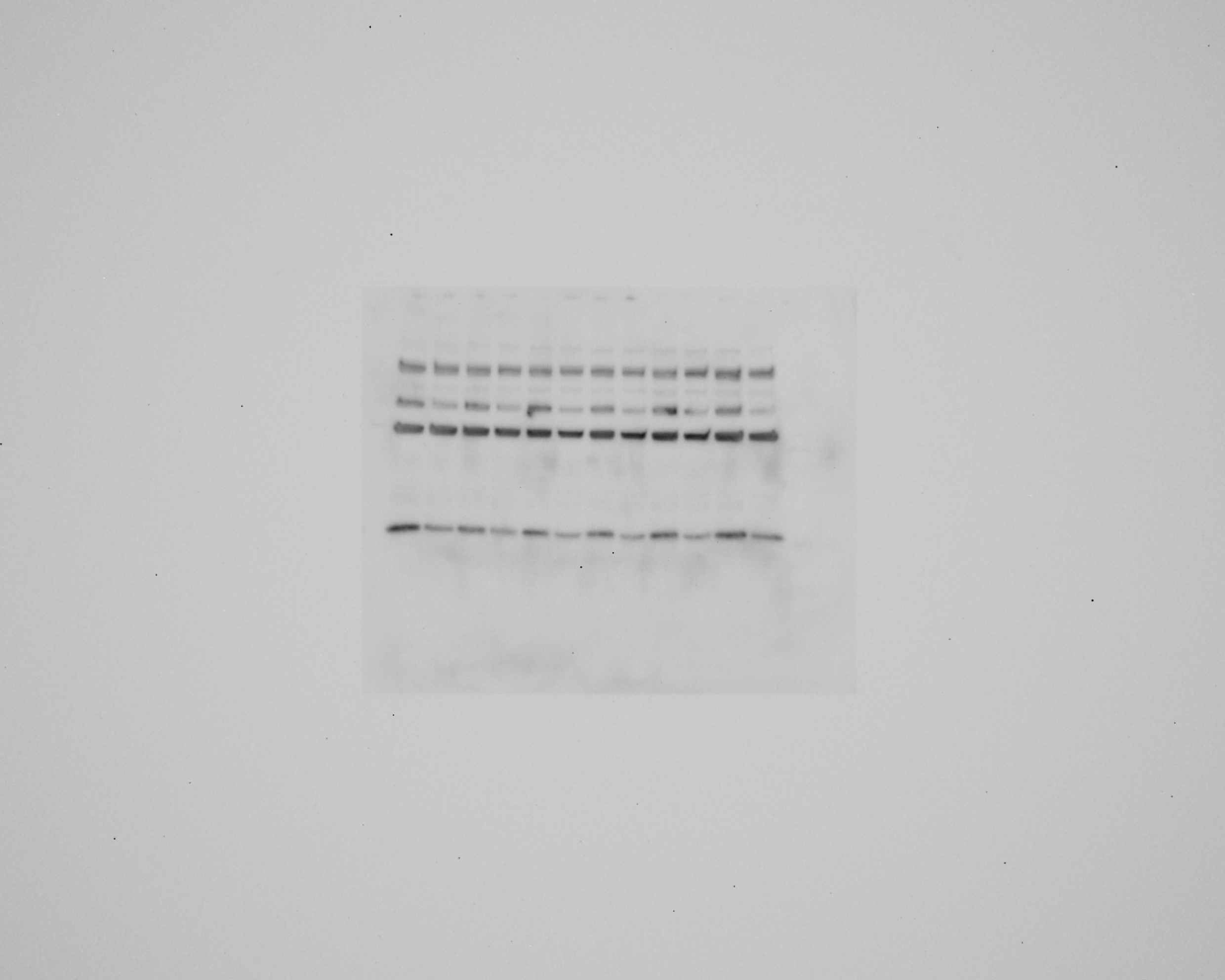

Supplement: Supplemental data [file mmc1.docx]
